# Reconstruction and Validation of a Genome-Scale Metabolic Model for the Filamentous Fungus *Neurospora crassa* Using FARM

**DOI:** 10.1371/journal.pcbi.1003126

**Published:** 2013-07-18

**Authors:** Jonathan M. Dreyfuss, Jeremy D. Zucker, Heather M. Hood, Linda R. Ocasio, Matthew S. Sachs, James E. Galagan

**Affiliations:** 1 Graduate Program in Bioinformatics, Boston University, Boston, Massachusetts, United States of America; 2 Department of Biomedical Engineering, Boston University, Boston, Massachusetts, United States of America; 3 Broad Institute of MIT and Harvard, Cambridge, Massachusetts, United States of America; 4 Tardigrade Biotechnologies, Jamaica Plain, Massachusetts, United States of America; 5 Institute of Environmental Health, Oregon Health & Science University, Portland, Oregon, United States of America; 6 Department of Biology, Texas A&M University, College Station, Texas, United States of America; The Pennsylvania State University, United States of America

## Abstract

The filamentous fungus *Neurospora crassa* played a central role in the development of twentieth-century genetics, biochemistry and molecular biology, and continues to serve as a model organism for eukaryotic biology. Here, we have reconstructed a genome-scale model of its metabolism. This model consists of 836 metabolic genes, 257 pathways, 6 cellular compartments, and is supported by extensive manual curation of 491 literature citations. To aid our reconstruction, we developed three optimization-based algorithms, which together comprise Fast Automated Reconstruction of Metabolism (FARM). These algorithms are: LInear MEtabolite Dilution Flux Balance Analysis (limed-FBA), which predicts flux while linearly accounting for metabolite dilution; One-step functional Pruning (OnePrune), which removes blocked reactions with a single compact linear program; and Consistent Reproduction Of growth/no-growth Phenotype (CROP), which reconciles differences between *in silico* and experimental gene essentiality faster than previous approaches. Against an independent test set of more than 300 essential/non-essential genes that were not used to train the model, the model displays 93% sensitivity and specificity. We also used the model to simulate the biochemical genetics experiments originally performed on *Neurospora* by comprehensively predicting nutrient rescue of essential genes and synthetic lethal interactions, and we provide detailed pathway-based mechanistic explanations of our predictions. Our model provides a reliable computational framework for the integration and interpretation of ongoing experimental efforts in *Neurospora*, and we anticipate that our methods will substantially reduce the manual effort required to develop high-quality genome-scale metabolic models for other organisms.

## Author Summary

Few organisms have been as foundational to the development of modern genetics and cellular metabolism as *Neurospora crassa*. Given the wealth of knowledge available for this filamentous fungus, the effort required to manually curate a high-quality genome-scale metabolic reconstruction would be daunting. To aid the reconstruction process, we developed three optimization-based algorithms. The first algorithm predicts flux while linearly accounting for metabolite dilution; the second algorithm removes blocked reactions with one compact linear program; and the third algorithm reconciles differences between *in silico* predictions and experimental observations of mutant viability. We have used these algorithms to develop the first genome-scale metabolic model for *Neurospora*. We have validated the accuracy of our model against an independent test set of more than 300 growth/no-growth phenotypes, and our model displays 93% sensitivity and specificity. Simulating the biochemical genetics experiments originally performed on *Neurospora*, we comprehensively predicted essential genes, nutrient rescues of auxotroph mutants and synthetic lethal interactions. With these predictions, we provide potential mechanistic insight into known mutant phenotypes, and testable hypotheses for novel mutant phenotypes. The model, the algorithms and the testable hypotheses provide a computational foundation for the study of *Neurospora crassa* metabolism.

## Introduction

First discovered as an orange mold infestation of Paris bakeries in 1843 [1], the filamentous fungus *Neurospora crassa* has become a model organism for eukaryotic biology and the cornerstone of a vibrant research community [2]. Work on *Neurospora* has led to essential discoveries in circadian rhythms [3], epigenetics [4], genome defense [5], mitochondrial biology [6], post-transcriptional gene silencing [7] and DNA repair [8]. Most famously, work in the 1940's by Beadle and Tatum led to the Nobel Prize-winning ‘one-gene-one-enzyme’ hypothesis that established the fundamental link between genes and proteins in all organisms [9], [10]. Work on *Neurospora* thus paved the way for modern genetics and molecular biology.

Of equal consequence, the work by Beadle and Tatum ushered in a new era in the study of biochemistry and cellular metabolism. The genetic facility of *Neurospora*, coupled with its ability to grow on minimal media, simplified the isolation of mutants with additional nutrient requirements. The first such auxotrophic mutants established the universal link among genes, enzymes, and the ordering of reactions in biosynthetic pathways. Work over subsequent decades led to a compilation of hundreds of such mutants, shedding light on most major biosynthetic pathways [11]–[13]. With the sequencing and annotation of the *Neurospora* genome [14], [15], these genetic data could be organized on a physical scaffold, genetic markers could be assigned to specific genes with predicted biochemical functions, genes could be assigned to previously orphaned biochemical reactions, and a global map of *Neurospora* metabolism could begin to emerge.

Genome-scale metabolic models have been constructed for over 100 organisms spanning bacteria to mammalian cells [16]. These network models capture information about all known metabolic reactions and the genes that encode enzymes for these reactions in a computationally structured manner. More than simply a catalog of reactions, network models capture biochemical relationships between reactions and pathways, afford a framework for integrating genomic measurements, and provide constraints for computational inference. One widely used method for computational inference using metabolic network models is Flux Balance Analysis (FBA) [17]. FBA calculates the flux of metabolites through a network under the assumption that metabolism is at steady state on the time-scales of interest. Using constraint-based modeling methods like FBA [18], it is possible to predict the growth rate of organisms under different conditions [19], the rate of production of metabolites of interest [20], the phenotypic consequences of gene knockouts, and the metabolic impact of different gene expression programs [21], [22]. Constraint-based methods are also being used to guide metabolic engineering efforts by calculating the modifications required to optimize the production of desired metabolites [23]–[26].

The wealth of genetic and metabolic data available for *Neurospora*, along with ongoing efforts to knock-out and phenotypically characterize all ∼10,000 genes in the genome [27], provides a strong foundation for the development of a genome-scale metabolic model. A metabolic model would, in turn, complement experimental efforts by integrating data from experiments on single genes into a coherent genome-wide metabolic framework, providing potential mechanistic insight into experimental phenotypic observations, and enabling the comprehensive modeling of perturbations that could not be feasibly performed in the lab. A genome-scale model is also a requirement for the rational and efficient use of *Neurospora* as a potential biofuels organism [28]–[32].

We report here the construction and validation of a high-quality genome-scale metabolic model for *Neurospora crassa*. To guide the process of model construction, we developed a novel suite of algorithms called *Fast Automated Reconstruction of Metabolism* (FARM). We validated the model against an independent gene essentiality test set, and achieved 93% sensitivity and specificity. We applied the validated model to comprehensively predict nutrient rescue of essential genes and synthetic lethal interactions. With these predictions, we provide potential mechanistic insight into known mutant phenotypes, and testable hypotheses for novel mutant phenotypes. More generally, the model provides a framework for integrating and interpreting ongoing experimental efforts that continue extend the rich history of biochemical research on *Neurospora*.

## Results

### Modeling process

**Figure 1:**
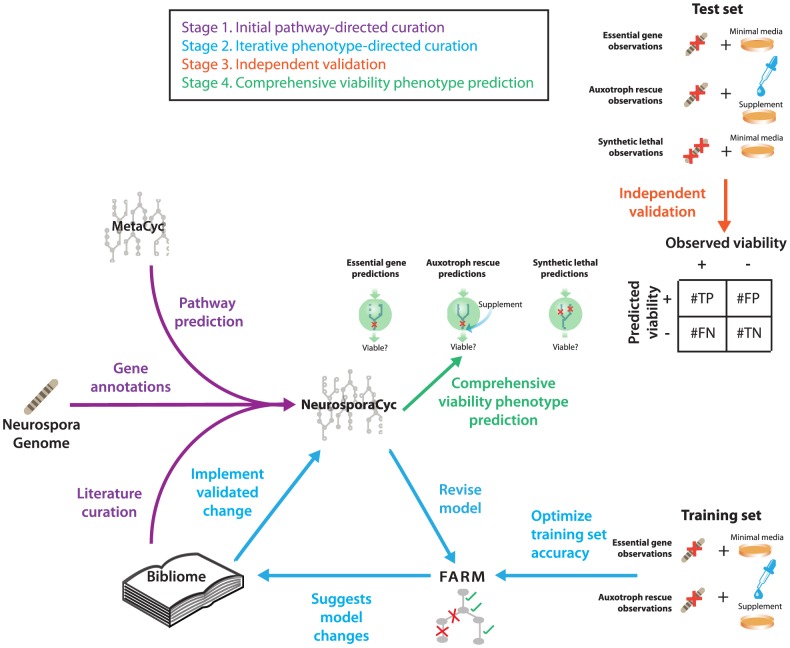
Modeling process. The process used for the reconstruction and validation of the metabolic model is described in four stages. In the first stage, *pathway-directed curation*, the genome sequence annotation [14], [15] , metabolic pathways derived from MetaCyc [34], [111] and experimental evidence from the *Neurospora* bibliome [37] were used to construct the first draft of the NeurosporaCyc Pathway/Genome database [112]. For the second stage, *iterative phenotype-directed curation*, we utilized FARM to suggest changes to the metabolic network based on a training set of experimentally observed growth phenotypes. These suggestions were reviewed manually, and accepted into the final model only if they were consistent with published experimental evidence. In the third stage, we *independently validated* the model based on a test set of experimentally observed viability phenotypes that were not utilized during model construction. In the fourth stage, we *comprehensively predicted* the phenotypes of all essential genes, nutrient rescues, and synthetic lethal interactions.

We reconstructed, validated and performed computational predictions with the *Neurospora* metabolic network model in a process consisting of four stages, as shown in Figure 1. Below we summarize the steps of the process, then we describe the optimization-based algorithms we developed to guide the process.

#### Stage 1: Pathway-directed curation

We integrated the *Neurospora* genome and literature to generate an initial draft of the metabolic network. This process was initiated by computing the probability that each enzyme activity is encoded in the genome sequence [14] using the *EFICA*z enzyme function predictor [33]. These predicted enzyme activities were then automatically assembled into experimentally elucidated pathways taken from MetaCyc [34] using the Pathologic pathway prediction algorithm [35]. Complementing this automated approach, we manually curated *Neurospora*-specific literature to identify experimentally determined enzymes, assign Gene Ontology terms to proteins, distinguish isozymes from enzyme complexes, catalog growth observations, and estimate the biomass composition [15], [27], [36]. Each assertion in the database was labeled with an evidence code to specify the type of experiment or computation performed to support its inclusion in the metabolic network [37].

#### Stage 2: Phenotype-directed curation

We iteratively improved the initial metabolic model with a manually curated training set of experimentally observed viability phenotypes on minimal and supplemented media. We used FARM on this set to suggest reaction additions/removals that would improve prediction accuracy. These changes were manually reviewed and accepted only if consistent with published experimental evidence.

#### Stage 3: Independent validation of model predictions

To confirm that the final model was not over-fit to a single training set and to ensure that the predictions of the model could generalize to new phenotypes, we validated the model using an independent test set of experimentally observed viability phenotypes.

#### Stage 4: Comprehensive viability phenotype prediction

We applied the final model to generate three sets of predictions. Firstly, we predicted the essentiality of all genes in our model. Secondly, we predicted which nutrient supplements would rescue a manually curated set of inviable mutants, and provided mechanistic explanations for each rescue. Thirdly, we systematically performed *in silico* double knockout experiments to predict synthetic lethal interactions. In all three cases, published observations were available that validated the accuracy of the predictions. The metabolic model extends these published observations in a manner that would be difficult experimentally by assaying a comprehensive set of conditions, providing novel testable hypotheses, and providing potential mechanistic insight into these predictions.

### FARM

A number of significant challenges remain in the reconstruction of high-quality genome-scale metabolic models [38]. Although bioinformatic methods exist that can automate the generation of draft metabolic models [39], extensive manual adjustment and literature curation remains essential for generating high-quality models. The assessment of model accuracy through independent empirical validation is also critical if the predictions of the model are to be trusted. Although a number of methods have been developed to aid in this task [40]–[51], substantial manual effort is also still required.

To facilitate the automation of metabolic network reconstruction, we developed three optimization-based algorithms, which together comprise *Fast Automated Reconstruction of Metabolism* (FARM). These algorithms are: *LInear MEtabolite Dilution Flux Balance Analysis* (limed-FBA), which predicts flux while linearly accounting for metabolite dilution; *Consistent Reproduction Of growth/no-growth Phenotype* (CROP), which reconciles differences between *in silico* and experimental gene essentiality faster than previous approaches; and *One-step functional Pruning* (OnePrune), which removes blocked reactions with a single compact linear program.

#### LInear MEtabolite Dilution Flux Balance Analysis (limed-FBA)

Flux balance analysis is a widely used method for predicting metabolic capabilities using genome-scale metabolic network models [17]. FBA represents a metabolic network by capturing the stoichiometries of constituent reactions in a stoichiometric matrix, *S*. The matrix *S* and the set of reaction constraints *lb* and *ub* define the set of all possible flux configurations *v* at steady state. By defining a metabolic objective function *c^T^v* that represents all the essential biomass components necessary for growth, linear programming can be used to predict whether the model supports growth under a given nutrient condition. The linear programming problem is:
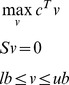
FBA can be used to predict gene essentiality by blocking reactions that correspond to the gene knockout and checking if the model can still support growth [18], [52](see Methods).

A known shortcoming of FBA is that it does not account for dilution of metabolites involved in active reactions [53]. These metabolites are referred to as *active metabolites*. Consequently, FBA can fail to require the biosynthesis of known essential compounds. For example, the *Saccharomyces cerevisiae* model [54] suffers its highest error rate in predicting growth of mutants deficient in quinone biosynthesis. The reason for this error is that FBA allows quinones to be recycled *in silico*, whereas biologically quinones must be replenished by *S. cerevisiae* to overcome their growth-associated dilution. To account for growth-associated dilution of active metabolites, we developed limed-FBA.

**Figure 2:**
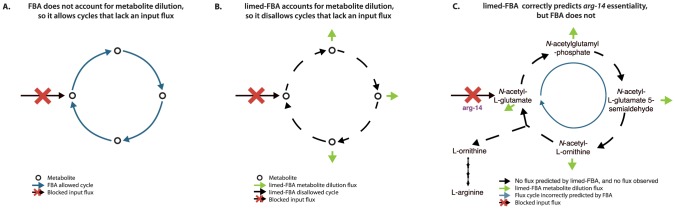
limed-FBA vs FBA. (A) FBA does not require an input flux for cycles because it does not account for dilution of metabolites that participate in active reactions. (B) limed-FBA requires an input flux for cycles to compensate for dilution of metabolites that participate in active reactions. (C) FBA fails to correctly predict *arg-14* gene essentiality because without an input flux, metabolite dilution prevents the isolated acetyl cycle compounds from being produced (side compounds not shown).

The limed-FBA method works by forcing active metabolites to dilute through an additional small dilution flux (see Methods). We illustrate the difference between limed-FBA and FBA in Figure 2. As shown, FBA does not account for metabolite dilution; it allows metabolic cycles that lack an input flux (Figure 2A). In contrast, limed-FBA forces dilution of active metabolites. This dilution necessitates a counteracting input flux, so limed-FBA disallows metabolic cycles that lack an input flux, as shown in Figure 2B. A specific example for *Neurospora* is shown in Figure 2C, which focuses on the gene *arg-14* that encodes acetylglutamate synthase. This enzyme acts as an input flux to arginine biosynthesis and is required for growth [55]. FBA uses the arginine biosynthesis pathway without input flux from acetylglutamate synthase, and thus incorrectly predicts *arg-14* is not essential. In contrast, limed-FBA forces dilution of the metabolites in the acetyl cycle, thus preventing these compounds from being produced without an input flux. As a consequence, limed-FBA correctly predicts that *arg-14* is essential.

A heuristic that is used in FBA to account for metabolite dilution is to add a small “drain” of diluted metabolites to the biomass composition. The issue with this heuristic is that it requires knowing a priori which metabolites are diluted, whereas limed-FBA determines which metabolites are diluted based on the flux. For example if this heuristic was used to add a metabolite in the cycle of Figure 2, such as *N*-acetyl-L-glutamate, to biomass, then FBA would correctly predict the essentiality of *arg-14*. However, then FBA would also predict that the *arg-14* knockout cannot be rescued by arginine. In fact, arginine does rescue Δ*arg-14* experimentally, as correctly predicted by limed-FBA.

Importantly, we designed limed-FBA as a linear program. Linear programs can be solved robustly and quickly, making limed-FBA a practical solution to account for metabolite dilution. An alternative method that has been developed is *Metabolite Dilution FBA* (MD-FBA) [53]. MD-FBA accounts for metabolite dilution by forcing a preset level of dilution for active metabolites. MD-FBA was shown to predict mutant growth more accurately than FBA [53], but it has two major drawbacks. (1) MD-FBA places a lower bound but no upper bound on dilution, so it effectively allows unlimited export of all metabolites, which is not biologically plausible; and (2) MD-FBA requires a computationally expensive *mixed integer linear program* (MILP), which severely limits its practicality [53].

#### Consistent Reproduction Of growth/no-growth Phenotype (CROP)

During stage 2 of our process, we iteratively improved the ability of the metabolic network model to predict gene knockout phenotypes. A number of computational algorithms have been described to maximize consistency between predicted and experimental growth/no-growth phenotypes [39]–[42], [44], [49]. These algorithms are typically designed to optimize a MILP, because they include binary variables to represent whether each reaction should or should not be included in the metabolic model. One such MILP-based algorithm is the Model SEED [39], , which is a fully-automated model reconstruction process for prokaryotes only. Another is GrowMatch [41], [42], which was designed to make small changes to models, such as adding or removing up to three reactions. One limitation of these approaches is that they do not account for the diverse evidence for reactions available for *Neurospora*, including enzyme function predictions, thermodynamic estimates, literature references, and pathway information, in a disciplined manner.

To quickly and accurately reconcile inconsistencies between predicted and experimental growth/no-growth phenotypes, we developed *Consistent Reproduction Of growth/no-growth Phenotype* (*CROP*). CROP solved inconsistencies while accounting for diverse evidence. This evidence included (1) whether we had manually curated a reaction with experimental evidence from the literature, (2) what pathways a reaction was part of, and whether these pathways were predicted to be in *Neurospora* [35], (3) thermodynamic estimates of Gibbs free energy, and (4) probabilistic estimates of enzyme function. This evidence was mathematically integrated on a probabilistic scale to assign each reaction a weight. Our approach to derive weights in a disciplined manner was motivated by the statistical maximum a posteriori estimator. Thus, the weights have a direct probabilistic interpretation: they depend on the product of the probabilities that the reaction is biochemically and thermodynamically plausible. These weights guided CROP's growth reconciliation toward metabolic network changes that were most consistent with available evidence. To achieve consistency when the model incorrectly predicts growth, CROP suggests reactions from the model to remove. To do this, CROP applies MILP. To achieve consistency when the model incorrectly predicts no-growth, CROP suggests reactions to add from a database of metabolic reactions, such as MetaCyc. A detailed comparison of CROP with previous methods is available in Text S3.

#### One-step functional pruning (OnePrune)

After iteratively applying CROP to improve the model, reactions can be present that cannot carry flux under any nutrient condition. These reactions are referred to as *blocked reactions*. The removal of blocked reactions is a process known as *functional pruning* [56]. Blocked reactions are often identified according to Flux Variability Analysis [57], [58], however this requires optimizing each reaction separately. An alternative approach [51] identifies blocked reactions by successive linear programs, although it is unknown in general how many LPs are necessary. We developed an algorithm to perform functional pruning with a single compact linear program, OnePrune. Conceptually, OnePrune is based on the optimization approach called *goal programming* [59]. We include a detailed account of OnePrune in Methods and in Discussion.

### Overview of *Neurospora* metabolic network model

Here we describe the results of our genome-scale metabolic reconstruction and its application as a predictive steady-state model. Following the accepted nomenclature [60] for naming metabolic models, we call the model *N. crassa* iJDZ836. *N. crassa* iJDZ836 is available in the Systems Biology Markup Language (SBML) in Dataset S1.

**Figure 3:**
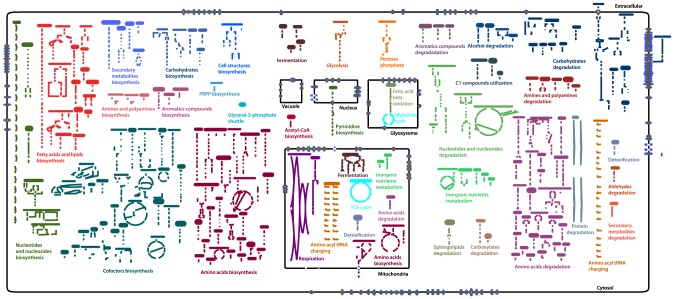
Metabolic overview of *Neurospora* crassa. The 257 metabolic pathways of *Neurospora* are divided into the 35 color-coded pathway classes. Biosynthetic pathways are displayed on the left, energy metabolism in the center, and degradation pathways are on the right. In addition to the cytosol and extracellular space, the model also contains 4 organelles: these are the vacuole, the nucleus, and the mitochondrion. The 299 transport reactions enable uptake and excretion of 137 metabolites and also exchange between the cytosol and each organelle.

The model contains 836 genes that encode 1027 unique enzymatic activities. Of these enzyme-catalyzed reactions, 694 are supported by experimental evidence from 491 publications addressing *Neurospora*-specific enzymes. This level of evidence compares favorably with other highly curated models [61]–[63] as shown in Table 1. In addition, 16 spontaneous reactions and 331 orphan reactions were included based on the literature [64]–[66]. Our model contains 737 chemically unique metabolites. Of these metabolites, 673 have a defined structure that permits estimates of Gibbs free energy [67], [68]. Using these Gibbs free energy estimates, 1046 biochemical reactions were thermodynamically constrained to be irreversible, while the remaining 328 were assumed to be reversible. Of the 294 biochemical reactions that were associated with multiple proteins, 47 were manually curated as being catalyzed by an enzyme complex; we assumed the rest were catalyzed by isozymes. There are 257 metabolic pathways in the model. Of these, 134 are biosynthesis pathways, 96 are degradation/utilization/assimilation pathways, and 27 are pathways involved in the generation of precursor metabolites and energy. An overview of the pathways is displayed in Figure 3 and a zoomable metabolic map of the pathways is shown in Figure S3.

**Table 1:** Comparison of curation level among selected metabolic models.

	*N.crassa* iJDZ836	*Aspergillus niger* iMA871	Yeast 5.0	*E.coli* iJO1366
Number of organism-specific citations	491	371	385	447
Coverage	47%	47%	37%	46%

Coverage is the percentage of enzyme-catalyzed reactions that are supported by organism-specific experimental evidence.

Cellular compartments in the model include the cytosol, the extracellular space, and 4 organelles: the glyoxysome, the vacuole, the nucleus, and the mitochondrion. The 299 transport reactions of the model enabled not only uptake and export of 137 metabolites, but also the exchange of metabolites between the cytosol and each organelle.

The model's growth objective was based on a modular biomass composition [69], [70]. Biomass modules were separately defined for DNA, RNA, amino acids, cell wall, lipids, sterols, essential cofactors, and secondary metabolites. This modular decomposition allowed for different goals in different applications of the model. For example, wild-type biomass contains substantial amounts of secondary metabolites, such as sphingolipids, ergosterol and carotenoids (which give *Neurospora* its characteristic orange color), so we included the secondary metabolites module in the biomass composition when predicting wild-type fluxes. On the other hand, secondary metabolites are not strictly required for viability, so we removed this module from the biomass composition when predicting viability.

The model quantitatively captures the growth rate of Neurospora. To illustrate this, we have plotted a range of glucose uptake rates against the model's predicted doubling times, and several data points extracted from the experimental literature [71]–[73] (Figure S4). The figure shows that our predictions closely match the experimental data.

### Model validation using gene essentiality

To validate the accuracy of the *N. crassa* iJDZ836 model, we manually curated a collection of mutant viability phenotypes from the literature. We split this collection into a training set, which we used with FARM to construct the model; and an independent test set, which we used to validate the final model. Both of these collections are available in Table S1. To simulate gene knockout experiments, we removed reactions from the model that depend on the gene, applied limed-FBA, and predicted whether or not the perturbed model could grow. We then compared experimental observations to the model's *in silico* predictions. Accuracy was measured as two quantities: sensitivity and specificity. Sensitivity was defined as the proportion of experimentally viable mutants that were predicted to be viable *in silico*. Specificity was defined as the proportion of experimentally inviable mutants that were predicted to be inviable *in silico*.

**Figure 4:**
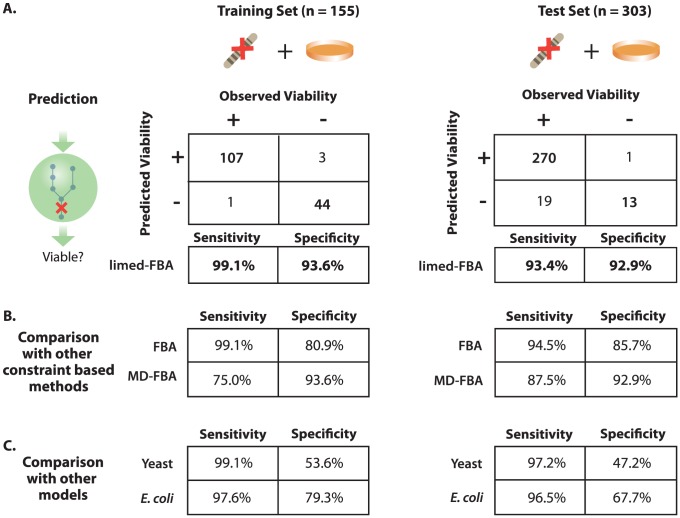
Minimal media gene essentiality predictions. We curated a collection of mutant viability observations on minimal media and separated the collection into a training set, where knowledge of the viability phenotype was used to improve the model; and a test set, where the viability phenotype was hidden from the model. (A) Training and test set mutant viability observations were used to measure the sensitivity and specificity of the limed-FBA gene knockout viability predictions. While some inconsistencies were due to model error, several were resolved in the model's favor, as discussed in the text. (B) Using the same model, training and test sets, limed-FBA outperforms FBA and MD-FBA. (C) For comparison, we display the mutant viability prediction accuracies of previously published FBA models for *S. cerevisiae* and *E. coli*. Prediction accuracies of experimentally observed viability phenotypes that were used to optimize the model are displayed on the left panel [41], [42]. Prediction accuracies of viability phenotypes that were not explicitly used to construct the model are displayed on the right panel [62], [63].

The final model's predictive accuracy using limed-FBA is shown in Figure 4A. On the training set, we correctly predicted growth in 107 of 108 experimentally viable gene knockouts (sensitivity = 99.1%), and we correctly predicted no-growth in 44 of 47 experimentally lethal mutants (specificity = 93.6%). On the test set, we correctly predicted 270 of 289 experimentally viable gene knockouts (sensitivity = 93.4%), and we correctly predicted 13 of 14 experimentally lethal mutants (specificity = 92.9%).

The final model's predictive accuracy on the test and training sets using FBA and MD-FBA is shown in Figure 4B. Both of these methods are generally outperformed by limed-FBA.

The differences between FBA and limed-FBA can reveal subtle issues with existing experimental data. For example, because FBA allows recycling of coenzyme A and the biosynthesis of coenzyme A requires *pan-2* [74], the essentiality of *pan-2* is missed by FBA. In contrast, limed-FBA correctly predicts essentiality of *pan-2*. Similarly, three other genes involved in coenzyme A biosynthesis (*NCU08925* and *pan-3*) and mitochondrial transport (*mic-30*) were predicted to be essential by limed-FBA, but not by FBA. Surprisingly, these three genes were in our test set of *non-essentials*. A potential explanation for these inconsistencies is that the function of these genes can be performed by an isozyme that was not captured in our model. In addition to these three genes, there is one more gene from our test set of non-essentials where limed-FBA predicted essentiality, but FBA predicted non-essentiality. This gene is *pab-1*. Like *arg-14* (Figure 2C), *pab-1* serves as input to a metabolic cycle, so *pab-1* is not required by FBA. However, consistent with limed-FBA's prediction, the *pab-1* gene was reported to be essential by Beadle and Tatum's original publication [9], [10].

In the left panel of Figure 4C, we compared the *N. crassa* iJDZ836 gene essentiality accuracy statistics on the training set to the reported accuracies *Escherichia coli* and *S. cerevisiae* models that were optimized using experimental observations of gene essentiality [41], [42]. In the right panel of Figure 4C, we compared the *N. crassa* iJDZ836 gene essentiality accuracy statistics on the test set to the accuracy statistics reported in the most recently published models of *E. coli* [63] and *S. cerevisiae* [62]. In all cases, the *N. crassa* iJDZ836 model prediction accuracies compare favorably, outperforming the specificities of the extensively trained models for *E. coli* and *S. cerevisiae*.

The prediction of gene essentiality for all genes in the model is available in Table S2.

#### Experimentally observed inviable mutants that were predicted viable

There were four inconsistencies where the model predicted viability and experimental data indicated lethality; these were *thi-4*, *ace-7*, and *ace-8* in the training set, and *arg-4* in the test set. The gene *thi-4* is required for its role in the thiamin diphosphate (TPP) biosynthesis pathway. However, this pathway includes the eukaryotic thiazole synthase, and to the best of our knowledge its reaction has not been characterized. Thus, this pathway was not included in the model. The incorrect prediction of the *ace-7* and *ace-8* mutants from the training set is likely due to regulatory effects, which our model does not capture. *ace-7* encodes a subunit of glucose-6-phosphate dehydrogenase, and it has been experimentally shown that glucose-6-phosphate dehydrogenase tightly controls NADPH regeneration [29], [75]–[78]. But since the model predicts that NADPH can be regenerated by many enzymes, we were unable to capture the essentiality of *ace-7*. The gene *ace-8* encodes pyruvate kinase, which is known to partially control glycolysis [79]. Thus, loss of *ace-8* could inhibit glycolysis *in vivo*, which would be lethal. But since the model predicts that pyruvate kinase's function can be circumvented by other enzyme activities, the model was unable to capture the essentiality of *ace-8*.

The only inconsistency in the test set where the model predicted viability was *arg-4*, which encodes acetylornithine-glutamate transacetylase. Upon closer examination, it turned out this mutant was experimentally observed to have some growth, albeit very little [80]. The model mechanistically explains viability by predicting that loss of acetylornithine-glutamate transacetylase activity can be compensated by acetylornithine deacetylase activity encoded by *arg-11*. Furthermore we do predict that the double knockout Δ*arg-4*Δ*arg-11* is synthetically lethal (see Figure S1).

#### Experimentally observed viable mutants that were predicted inviable

There was only one inconsistency in the training set where the model predicted lethality and the experimental data indicated viability, and this prediction revealed an error in the underlying experimental data. The experimental phenotyping for the Δ*erg-14* knockout indicated growth, and hence we included this gene in the non-essential training set. In contrast, the model predicted that the Δ*erg-14* knockout was blocked in the production of mevalonate, which is a necessary precursor for the sterol component of biomass. Moreover, previous attempts to phenotype temperature-sensitive mutants of *erg-14* revealed severe morphological defects that were expected to be lethal in the full knockout [81]. Driven by these inconsistencies, a re-examination of the Δ*erg-14* knockout strain revealed this mutant used was in fact a heterokaryon that retained a copy of the *erg-14* gene rather than a homokaryon that contained no *erg-14* gene, as originally thought. Thus the predictions of the model were sufficient to correct an error in metadata associated with a publically available knockout strain.

We identified 19 inconsistencies in the test set where the model predicted inviability and experimental data [82] indicated viability. We describe these in Text S1.

### Prediction of nutrient rescue

To validate the ability of the *N. crassa* iJDZ836 model to predict nutrient supplements that would rescue auxotroph mutants, we manually curated a collection of nutrient rescue conditions from the literature. We split this collection into a training set, which we used with FARM to construct the model; and an independent test set, which we used to validate the final model. Both of these collections are available in Table S1. The predictions of nutrient rescues are available in Table S2. To simulate nutrient rescue experiments, we took a mutant that was predicted to be inviable on minimal media, supplemented the media with different nutrients, and applied limed-FBA to predict whether or not the mutant could grow in the supplemented media. We then compared experimental observations to the model's *in silico* predictions.

**Figure 5:**
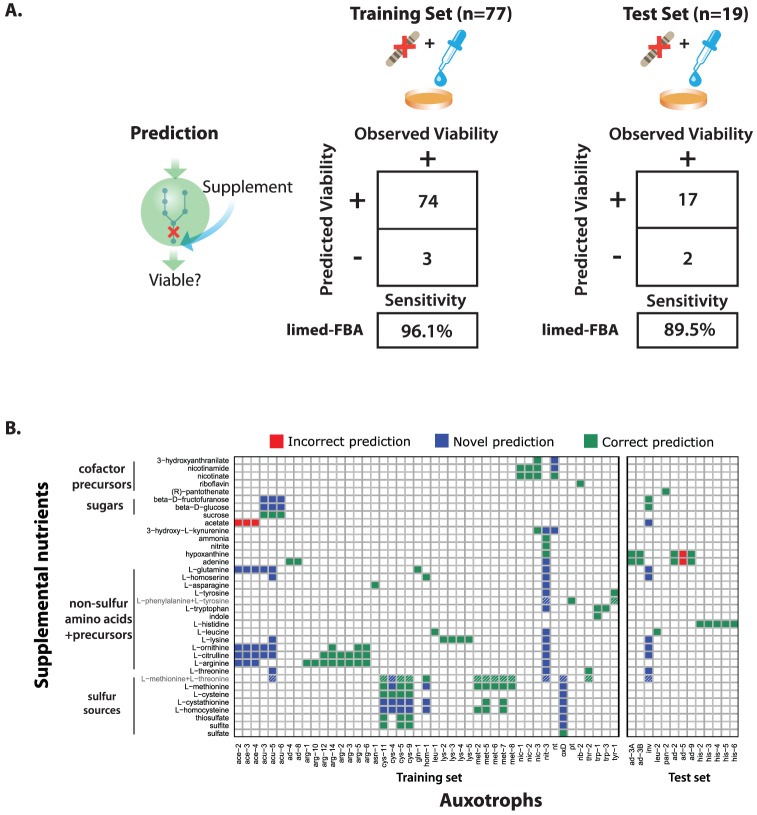
Prediction of nutrient rescue. We curated a collection of conditions in which an auxotroph was rescued when minimal media was supplemented with a nutrient. We separated the collection into a training set, where knowledge of the rescue phenotype was used to improve the model, and a test set, where the rescue phenotype was hidden from the model. Because we only collected data on which nutrients rescued the auxotrophs, we could only measure sensitivity, not specificity. (A) Tables showing the sensitivity of limed-FBA predictions on nutrient rescue training and test sets. (B) Heatmap showing the growth phenotype of each mutant when minimal media is supplemented with each nutrient used in the training and test sets. Only mutants whose minimal media gene essentiality was correctly predicted are included. The minimal media used was Vogel's with sucrose as the carbon source except in the following cases: *acu-3,5,6* genes are essential when acetate is the sole carbon source; *oxD* is essential when D-methionine is the sole sulfur source; *nit-3* is essential when nitrate is the sole nitrogen source. Green squares indicate that the model's predictions were consistent with experiment; red squares indicate that the model failed to correctly predict growth; blue squares indicate potentially novel rescues; white squares indicate predictions of non-rescue. Striped squares show that the multi-substrate case does not contain additional information beyond the single-substrate case, e.g. methionine is predicted to rescue the *cys-4* mutant, so methionine+threonine is also predicted to rescue *cys-4*.

Of the 77 experimentally observed nutrient rescue conditions that we used as a training set, the model correctly predicted 74 (sensitivity = 96.1%)(Figure 5A; left panel). On the independent test set of 19 nutrient rescue conditions, the model correctly predicted 17 (sensitivity = 89.5%)(Figure 5A; right panel).

#### Experimentally observed nutrient rescues that were not predicted

In the training set, the only auxotrophs we were unable to correctly rescue were due to condition-specific regulation that our model does not capture. According to experimental observation, mutants in *ace-2*, *ace-3* and *ace*-*4* can grow in acetate minimal media, because the enzymes in the glyoxysome are induced when extracellular acetate is present in the medium [83]. Conversely, *ace-2*, *ace-3* and *ace-4* mutants cannot grow in sucrose minimal media, even though sucrose can be converted to acetate intracellularly, because the glyoxysomal enzymes are not expressed in this condition [1]. Because the acetate-dependent regulation of the glyoxysome is not included in our model, we could not successfully predict both their inviability in sucrose minimal media and their rescue by acetate supplementation.

In the test set, we were unable to correctly rescue *ad-5* by hypoxanthine or adenine due to large amounts of experimentally observed accumulation of AICAR [84], which neither FBA nor limed-FBA allow. When we relaxed the *in silico* constraint on intracellular accumulation of AICAR, the hypoxanthine and adenine rescues of *ad-5* were correctly predicted.

#### Mechanistic insight of nutrient rescue

Simulating the biochemical genetics experiments originally performed on *Neurospora*, we predicted 175 nutrient rescues of 58 auxotroph mutants. These predictions are shown in Figure 5B. In addition to predicting the nutrient conditions that rescue selected mutants, the model also provides a potential mechanistic explanation for the rescue.

**Figure 6:**
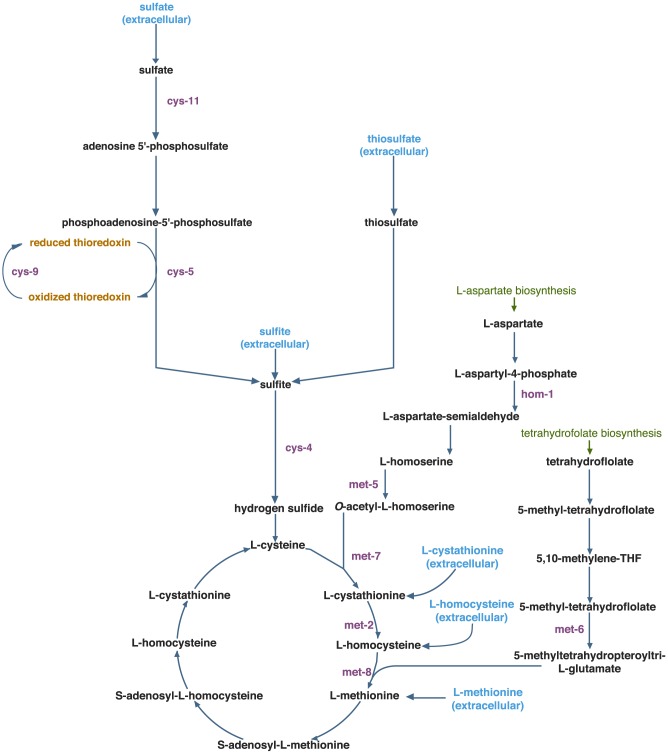
Mechanistic insight into the nutrient rescue of cysteine and methionine metabolism. The model correctly predicts that *cys-5*, *cys-9*, and *cys-11* mutants can be rescued when the downstream nutrients sulfite and thiosulfate are provided in the media. Similarly, the model correctly predicts that *met-2*, *met-5*, *met-6*, *met7* and *met-8* mutants are rescued by L-methionine; *met-2*, *met-5* and *met-7* mutants are rescued by L-homocysteine; and *met-5* and *met-7* mutants are rescued by L-cystathione. The model makes the potentially novel predictions that *hom-1* and all *cys* mutants can be rescued by the downstream supplements L-cystathione, L-homocysteine and L-methionine. The model also makes the potentially novel prediction that *cys-4* is not rescued by either upstream nutrient supplements sulfite or thiosulfate.

Most of the predictions can be explained by the general principle that supplementing a metabolic pathway downstream of a knockout will often rescue the mutant, while supplementing the pathway upstream of the knockout typically does not [9]. This is illustrated in Figure 6, where the model correctly predicts that *cys-5*, *cys-9*, and *cys-11* mutants can be rescued when the downstream nutrients sulfite and thiosulfate are provided in the media [85]. Similarly, the model correctly predicts that *met-2*, *met-5*, *met-6*, *met-7* and *met-8* mutants are rescued by L-methionine; *met-2*, *met-5* and *met-7* mutants are rescued by L-homocysteine; and *met-5* and *met-7* mutants are rescued by L-cystathione [86], [87].

This principle provides a testable hypothesis for the novel predictions that *hom-1* and all *cys* mutants can be rescued by the downstream supplements L-cystathione, L-homocysteine and L-methionine (see Figure 6). Conversely, we also predict that the *cys-4* mutant is not rescued by either upstream supplements sulfite or thiosulfate.

**Figure 7:**
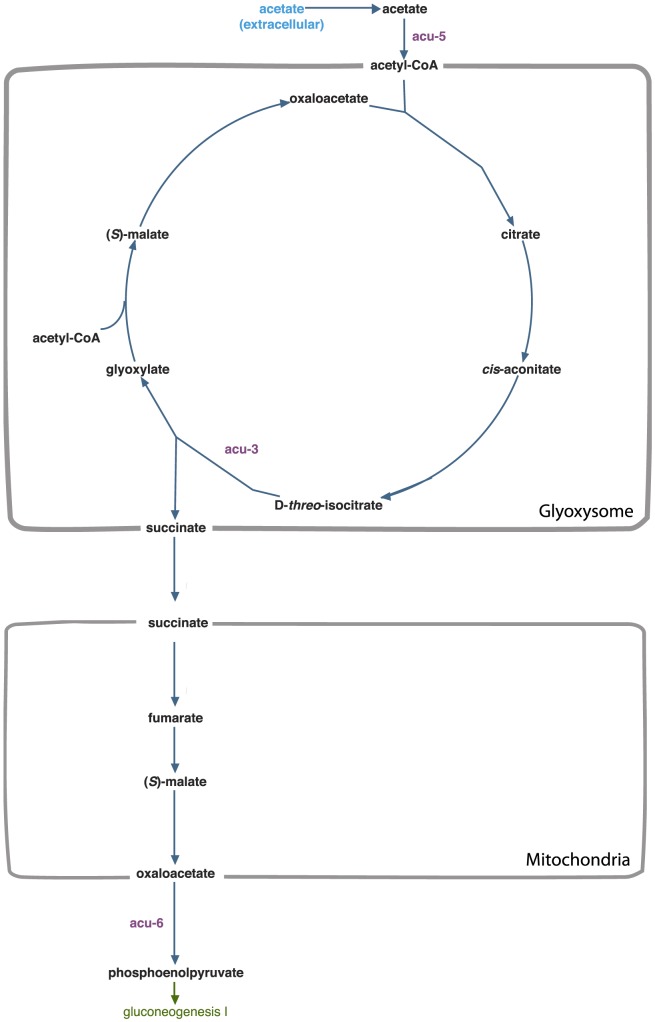
Connection between glyoxylate cycle and gluconeogenesis reveals mechanistic insight into the nutrient rescue of *acu* mutants. *acu-3*, *acu-5* and *acu-6* mutants are known to be lethal when acetate is the sole carbon source, because the glyoxylate cycle is blocked [88]. We correctly predict these mutants can be rescued by sucrose, and we additionally predict they can be rescued when supplemented with fructofuranose and glucose, because the enzymes encoded by *acu-3*, *acu-5*, and *acu-6* are upstream of these sugars in the gluconeogenesis pathway.

Using this principle, Figure 7 shows how the nutrient rescue of *acu* mutants can be explained by examining the connection between the glyoxylate cycle and gluconeogenesis. *acu-3*, *acu-5* and *acu-6* mutants are known to be lethal when acetate is the sole carbon source, because the glyoxylate cycle is blocked [88]. We correctly predict these mutants can be rescued by sucrose [89], and we additionally predict they can be rescued when supplemented with fructofuranose and glucose, because the enzymes encoded by *acu-3*, *acu-5*, and *acu-6* are upstream of these sugars in the gluconeogenesis pathway.

**Figure 8:**
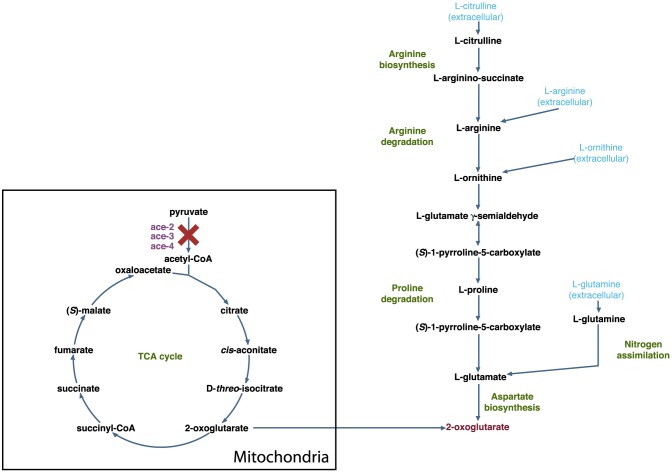
Supplementing with nutrients in alternate pathways can rescue some mutants. The model makes the novel prediction that *ace-2*, *ace-3*, and *ace-4* mutants (purple) in the TCA cycle can be rescued by supplementing minimal media with L-citrulline, L-arginine, L-ornithine, or L-glutamine (light blue) because each of these nutrients provide an alternate route via amino acid pathways to the essential metabolite 2-oxoglutarate (red).

Some novel nutrient rescue predictions can be explained by the existence of an alternate pathway from the nutrient to an essential metabolite that could not otherwise be produced. For example, Figure 8 shows that the *ace-2*, *ace-3*, and *ace-4* gene products are all components of the pyruvate dehydrogenase complex, which synthesizes acetyl-CoA from pyruvate, and that this activity leads to the production of the essential metabolite 2-oxoglutarate (α-ketoglutarate) via the TCA cycle. The model also predicts that *ace-2,3,4* mutants can be rescued by L-citrulline, L-arginine, L-ornithine, and L-glutamine, because each of these nutrients can produce 2-oxoglutarate via amino acid degradation pathways.

### Prediction of synthetic lethal interactions

Pairwise synthetic lethality arises when two mutants with single gene knockouts are viable, but the double knockout mutant is inviable. Synthetic lethality can reveal cross-pathway dependencies that provide valuable insights into metabolism at the genome scale, but an experimental approach to comprehensively perform double knockouts to identify synthetic lethals is currently infeasible for *Neurospora*. Computational models provide a mechanism to rapidly and comprehensively test all interactions as a way to prioritize subsequent experimental verification. To predict synthetic lethality using the model, we simulated all pairs of knockouts of non-essential genes and predicted viability on minimal media.

**Figure 9:**
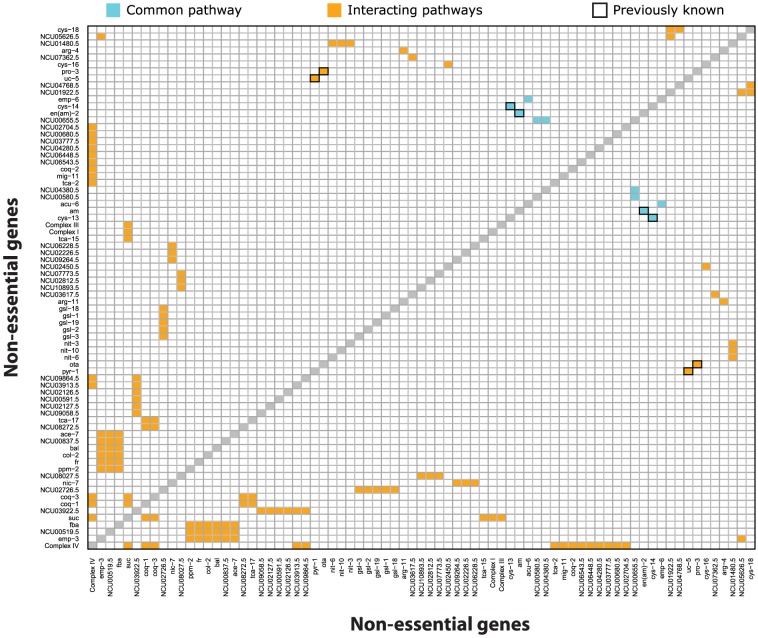
Synthetic lethality interaction map. This gene-by-gene interaction map shows synthetic lethal predictions on Vogel's minimal media, except the double mutant *pyr-1*:*uc-5* is on Vogel's+uracil. Shown are non-isozyme pairs, except the previously known isozyme pair *cys-13:cys-14*. If both synthetic lethal genes of a pair are in a common pathway, the square is cyan; if they are in interacting pathways, then it is colored orange. Validated synthetic lethal predictions have a black border.

Of the roughly 700,000 double knockouts in the metabolic model, the model predicted 230 to be synthetically lethal on minimal media. The mechanisms underlying these predicted synthetic lethal interactions fall into three classes: those that encode isozymes of a common reaction, those that encode enzymes of a common pathway, and those that encode enzymes of interacting pathways. This list contains 22 isozyme pairs, 4 gene pairs in the same pathway, and 204 gene pairs in interacting pathways. All of these pairs are tabulated in Table S2. The non-isozyme gene pairs and a previously known isozyme pair are displayed in a symmetric interaction map in Figure 9. This interaction map classifies each synthetic lethal pair by whether or not the two genes are in a common pathway or interacting pathways.

#### Mechanistic insight of synthetic lethal interactions

To validate the ability of the model to predict synthetic lethality, we manually curated a small number of experimentally observed synthetic lethal interactions. Some of these interactions involved the *arg-2* mutant, which is known to be “leaky” [55]. We used the remaining synthetic lethal interactions to validate our results. Of these 5 experimentally observed synthetic lethal interactions, the model correctly predicts 4.

The single known interaction not predicted by the model is for the double mutant *pho-4*:*pho-5* [90]. Both of these genes are high-affinity phosphate transporters, but the model also includes a known low-affinity phosphate transporter. The model treats each of these transporters equally, because it lacks kinetics, so it predicts the *pho-4*:*pho-5* mutant is viable.

**Figure 10:**
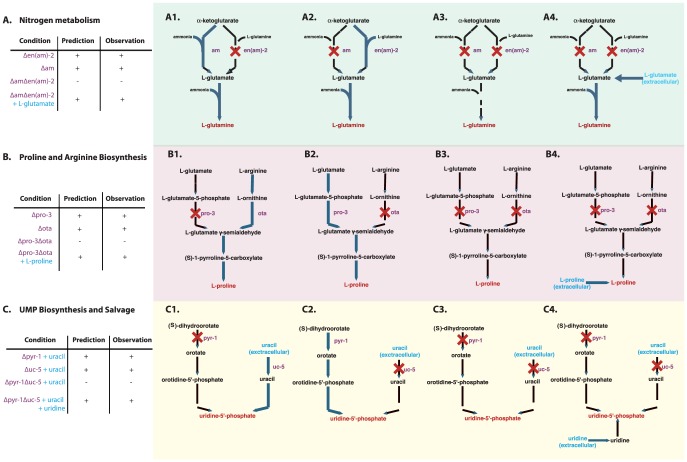
Mechanistic insight into three experimentally validated synthetic lethal auxotrophs and their nutrient rescue. (A) The nitrogen assimilation pathway contains two alternate routes that convert α-ketoglutarate into the essential metabolite L-glutamine (red). (A1) The *en(am)-2* mutant is viable, because α-ketoglutarate can be aminated to L-glutamate via *am*. (A2) The *am* mutant is viable, because α-ketoglutarate and L-glutamine can be converted to 2 L-glutamate via *en(am)-2*. (A3) The double mutant *am*:*en(am)-2* is lethal when ammonium is the nitrogen source because both routes to L-glutamine are blocked, but (A4) can be rescued when the media is supplemented with L-glutamate (A4). (B) The only two routes for the synthesis of the essential metabolite L-proline are through arginine degradation and proline biosynthesis. (B1) The *pro-3* mutant is blocked in proline biosynthesis, but can obtain L-proline through arginine degradation. (B2) The *ota* mutant is blocked in arginine degradation, but can obtain L-proline through proline biosynthesis. (B3) The double mutant *pro-3*:*ota* is blocked in both routes, but can be rescued when the nutrient media is supplemented with L-proline (B4). (C) There are only two biosynthetic routes to the essential metabolite uridine-5′-phosphate. (C1) The *pyr-1* mutant can still obtain uridine-5′-phosphate from extracellular uracil, and the *uc-5* mutant can obtain uridine-5′-phosphate from (S)-dihydroorotate (C2), but the *pyr-1*:*uc-5* double mutant is blocked in both routes (C3). However, it can be rescued when the nutrient media is supplemented with uridine through its conversion to uridine-5′-phosphate in the pyrimidine salvage pathways (C4). Side compounds not shown.

Figure 10 shows three known synthetic lethal interactions that we correctly predict. Figure 10A provides an example of synthetic lethality arising from mutations in a common pathway. The nitrogen assimilation pathway fixes the nutrient nitrate into the essential metabolite glutamine. Both the *am* and *en(am)-2* mutants are viable, because they represent the two alternate routes to synthesize glutamine. However, the double mutant *am:en(am)-2* is blocked in both routes, so it is inviable, unless the nutrient media is supplemented with glutamate.

Figure 10B provides an example of synthetic lethality arising from mutations in two interacting pathways. The pathways for proline biosynthesis and arginine degradation both synthesize glutamate-semialdehyde, which is a precursor to the essential metabolite L-proline. Both the *pro-3* (glutamate-5-semialdehyde dehydrogenase) mutant in proline biosynthesis, and the *ota* (acetylornithine transaminase) mutant in arginine degradation are viable, because they represent the two alternate routes to synthesize L-proline. However, the double mutant *pro-3*:*ota* is blocked in both routes, so it is inviable, unless the nutrient media is supplemented with L-proline.

Figure 10C provides another example of synthetic lethality arising from two interacting pathways. On uracil-containing media, the uridine-5′-phosphate synthesis pathway and the path from uracil to uridine-5′-phosphate (UMP) both synthesize the essential UMP. So on uracil-containing media, both *pyr-1* (dihydroorotate dehydrogenase) in uridine-5′-phosphate synthesis and *uc-5* (uracil permease) are viable, because they represent the two alternate routes to synthesize UMP. Consequently, the double mutant *pyr-1*:*uc-5* on uracil-containing media is blocked in both routes, so it is inviable. Further, the *pyr-1*:*uc-5* mutant can be rescued by uridine, since uridine can be phosphorylated to UMP.

The novel predictions of synthetic lethality provide testable hypotheses for further experimentation. For example, the model predicts synthetic lethality between *NCU02726* (ethanolamine kinase) and *gsl-3* (3-dehydrosphinganine reductase). The products of these two genes catalyze reactions in two different pathways that lead to the production of the essential metabolite phosphoryl-ethanolamine. Ethanolamine kinase converts ethanolamine to phosphoryl-ethanolamine in a single reaction. Without ethanolamine kinase, the model predicts that phosphoryl-ethanolamine must be produced from the sphingolipid metabolism pathway. This requires the activity of *gsl-3*, which is an upstream member of this pathway.

Figure S2 illustrates the potential mechanism underlying the predicted synthetic lethality between the *suc* gene (pyruvate carboxylase) and subunits of mitochondrial complex I (NADH:ubiquinone oxidoreductase). Under normal circumstances, NADH produced in the mitochondrial TCA cycle is oxidized to NAD in the electron transport chain via NADH:ubiquinone oxidoreductase. Yet, the loss of complex I is known to be non-lethal in *Neurospora* [91]. This places a metabolic burden on the rest of mitochondrial metabolism to oxidize the NADH. The model predicts that in the absence of complex I, NADH is oxidized by malate dehydrogenase in the reverse direction of the normal TCA cycle flux. To maintain this flux, the mitochondrion requires a steady source of oxaloacetate, which can only be supplied from pyruvate carboxylase, encoded by *suc*. This synthetic interaction is particularly noteworthy, because *S. cerevisiae* lacks complex I, making *Neurospora* a prime model for studying its interactions.

## Discussion

Building on its long history as a genetic model organism for biochemical genetics and cellular metabolism, we report here the first genome-scale metabolic network model for *Neurospora crassa*. We assessed the *Neurospora* metabolic model's ability to predict the impact of gene deletions, nutrient supplements that would rescue essential gene deletions, and synthetic lethal interactions. In each case, computational predictions were validated against a curated dataset of experimentally observed mutant viability phenotypes. Importantly, to ensure that our model was not over-fit, we separated the experimental data into a training and test set. Whereas training data was used in the development of the model, the test set was reserved to assess the accuracy of the final model. The final accuracy assessment was thus independent of any data used during model training.

The prediction of the growth phenotype of gene deletions is a canonical test of metabolic model accuracy and a useful benchmark for assessing the quality of different models [92]. The accuracy of our model compares favorably to extensively curated models such as *S. cerevisiae* and *E. coli*. Moreover, at 93% sensitivity and specificity on a test set of 303 phenotyped gene knockouts, the *Neurospora* model displays high absolute accuracy that lends confidence to the ability of the model to make accurate novel predictions.

The *N. crassa* iJDZ836 model also demonstrates high accuracy in predicting the ability of different nutrients to rescue essential gene knockouts and in predicting synthetic lethal interactions. In the former case, the model displays nearly 90% accuracy on an independent test set of nutrient rescue experiments. In the latter case, we were only able to curate a handful of experimentally verified synthetic lethal interactions. Nonetheless, although no synthetic lethal data was used during model training, the model was able to correctly identify four out of five known synthetic lethal interactions.

Genome-scale metabolic models complement experimental investigations, and one role of metabolic modeling is to rapidly generate and prioritize testable predictions that can be used to guide subsequent experimentation. As important as the predictions themselves, metabolic models also provide potential mechanistic explanations for the results. The explanations provide an important check on the overlying predictions. During the validation of models, these explanations ensure that not only are correct answers given, they are given for valid underlying reasons. For novel predictions, mechanistic explanations can provide potential insight into the results as well as tangible avenues to experimental validation. To illustrate the last point, in Text S4 and Figure S5 we simulate the observed physiological effect of oxygen limitation on ethanol production when grown on xylose. Therefore, our model can be used to simulate perturbations that optimize ethanol yield, which can then be verified experimentally.

As with all previous modeling efforts, errors in predicting known experimental results highlight limitations in either the model itself or the modeling framework. In terms of the model, the quality will only be as good as the information that was used to develop it. In the case of *Neurospora*, the extraordinarily rich literature for this well-studied model organism was the foundation that enabled a model to be generated that performed with high accuracy. Nonetheless, certain areas of the model remain less well developed, and one value of model construction is the objective measure it can provide on the relative information available for different aspects of metabolism. This can be used to target areas that are less well understood. For example, the substrates of certain reactions in the thiamin diphosphate and neurosporaxanthin biosynthesis pathways and the fate of the end-product in the histidine degradation pathway cannot be included with confidence in any metabolic model, because they are open biochemical questions [93].

More generally, the constraint-based modeling framework we used here is known to suffer from certain limitations. As with similar models, this accounts for a significant portion of the prediction errors in the *Neurospora* model. In particular, our model does not account for regulation of either enzyme expression or activity. These factors sometimes acted in combination. An illustrative example is *gln-1* and *gln-2*, which code for the alpha and beta subunit, respectively, of glutamine synthetase [94]. Our model requires both subunits for enzyme catalysis. However, it was experimentally shown that concentration of extracellular ammonium regulates this enzyme's subunit composition, which can include both subunits, only alpha subunits, or only beta subunits [95]. This metabolic complexity highlights the need for the future incorporation of kinetics and regulation.

In one instance, however, a prediction initially thought to be an error provided the means to identify an issue with an experimentally observed knockout. The viability phenotype experiment for Δ*erg-14* was performed on a knockout strain originally designated as a homokaryon. Experimental observations of this strain revealed a normal growth phenotype. In contrast, the model predicted that the Δ*erg-14* mutant was blocked in the production of mevalonate, which is a necessary precursor for the sterol component of biomass. Moreover, previous efforts to phenotype temperature-sensitive mutants of *erg-14* revealed severe morphological defects that were expected to be lethal in the full knockout [81]. Driven by these inconsistencies, a re-examination of the Δ*erg-14* knockout revealed that the mutant used was in fact a heterokaryon. This prediction, in effect, served as a blind control that highlighted the predictive value of the model.

The construction of genome-scale metabolic models remains a daunting task. Even aided by sophisticated tools for the management and visualization of pathway knowledge, a metabolic reconstruction still requires substantial manual review of the corresponding literature [38]. Moreover, it is desirable that the model construction process be guided by objective and quantitative measures of predictive accuracy. Incorporating this requirement into the model generation process increases the complexity of the task by requiring iterative cycles of data curation, model improvement, and accuracy assessment. To facilitate the process of model improvement, a number of tools have been developed [39]–[47], [49], [51]. We contribute to this set of tools with the development of a set of optimization-based algorithms, which together comprise *Fast Automated Reconstruction of Metabolism* (FARM).

Two of the three FARM algorithms specifically facilitate the process of model construction. *Consistent Reproduction Of growth/no-growth Phenotype* (CROP) assists in automating the process of adding and subtracting reactions from a model to improve predictive accuracy. CROP integrates diverse evidence for pathways into a probabilistic framework that assigns a weight to each reaction associated with the likelihood that the reaction is present in the network. These weights are then used to guide the selection of reactions to add or remove. Previous methods to achieve *in silico* growth used mixed integer linear programming (MILP), and thus required substantial compute time [39], [42], [44]. For CROP, we utilize LP relaxation, which is faster by orders-of-magnitude. Additional details for this algorithm along with comparisons to GrowMatch [41], [42] and Model SEED [39], [40] are available in Text S3.

OnePrune was developed to efficiently solve the problem of removing reactions that are blocked. OnePrune utilizes the goal programming optimization framework to achieve multiple competing objectives. The advantage of this framework is that once an individual objective is achieved, the optimization can pursue other objectives. OnePrune's goals are to send flux through as many reactions as possible, so once a reaction has achieved a nonzero flux, OnePrune is free to pursue flux through other reactions. Thus, OnePrune identified which reactions could carry flux with a single compact linear program.

The third FARM algorithm, limed-FBA, is an enhancement to the FBA method that improves predictive accuracy. limed-FBA accounts for the dilution of active metabolites that is ignored by FBA (Figure 2B), so limed-FBA is able to correctly identify the essentiality of reactions that are typically missed by standard FBA. For example, FBA predicted that *pab-1* was not essential, because *pab-1* serves as input to a metabolic cycle; however, limed-FBA predicted that *pab-1* was essential. In fact, essentiality of *pab-1* was shown by the original experiment of Beadle and Tatum on *Neurospora crassa* [9], [10].

## Methods

### Metabolic reconstruction protocol

The metabolic reconstruction of *Neurospora crassa* was performed in accordance with previously described protocols [96], [97], and is detailed in Text S2.

### Creation and curation of NeurosporaCyc

From the *Neurospora crassa* genome assembly NC10, gene boundaries were predicted using the Calhoun annotation system [14]. For all enzymes, we obtained the probability that the enzyme catalyzes a particular biochemical reaction, characterized by its Enzyme Commission (EC) number, from the enzyme function prediction software *EFICA*z [33].

We used the Pathway Tools software suite [93] to create the NeurosporaCyc Pathway/Genome Database (PGDB) and to manage curated data. We added functional gene annotations with associated Gene Ontology (GO) terms [36] and literature citations that were manually curated by the Community Annotation Project [27]. EC numbers, GO terms, and functional annotations were used as input to Pathologic [35] to automatically infer pathways from MetaCyc. These pathways comprised the initial NeurosporaCyc PGDB. Reaction directions were based on the Gibbs free energy predictions using the group contribution method [67]. Enzyme complexes were manually curated using the Pathway Tools protein complex editor and evidence from the *Neurospora* literature [93]. Transporters were automatically predicted from the genome sequence by the Transporter Automatic Annotation Pipeline (TransAAP) from TransportDB [98], and from the genome annotation using the Transport Identification Parser (TIP) [99]. Cellular compartment information was described using the Cellular Component Ontology (CCO), available at http://bioinformatics.ai.sri.com/CCO/. *Neurospora*-specific enzyme kinetics, allosteric regulation, biomass composition, and growth media were added during manual curation of the experimental evidence in the literature.

Before the NeurosporaCyc PGDB could be used to generate a working model, a number of data-cleaning steps were performed. Reactions were mass balanced, and the individual metabolites were protonated to the intracellular pH of 7.2 [100]. For reactions containing compound classes (e.g. “an alcohol”), the compound class was replaced by its instances (e.g. “ethanol”) that render the equation mass-balanced. Polymerization pathways such as fatty acid beta oxidation were either lumped into a summary reaction, or instantiated into a chain of individual polymerization steps. For polymerization reactions, we specified an arbitrary maximum polymer size *n* and created a lumped reaction that was stoichiometrically equivalent to *n* steps of the polymerization. The NeurosporaCyc Pathway/Genome Database can be downloaded from the PGDB registry at http://biocyc.org/registry.html and is available online at http://neurosporacyc.broadinstitute.org.

### Curation of growth rate data

To curate our growth rate data, we identified several manuscripts that included glucose concentrations for Neurospora grown on glucose minimal medium where doubling times could be inferred from growth curves [71] or were given [72], [73]. The glucose concentrations were converted to glucose uptake rates using data for the derepressed system in Figure 1 of Schneider and Wiley [101].

### Curation of viability phenotype data

To curate our viability phenotype data, we primarily relied on two resources. The first was *The Neurospora crassa e-Compendium*, curated by Alan Radford. The e-Compendium contains >2,400 citations and >3,000 gene entries. Numerous gene entries have associated mutant phenotypes extracted from the literature. In some cases, these phenotypes also include supplements that rescue no-growth mutants. Because this resource primarily lists phenotypes from mutants that are not knockouts, viability of a mutant could be due to non-essentiality of a mutated gene or to partial efficacy of a mutated enzyme. Thus, this resource did not clearly identify non-essential genes. The second resource was knockouts from the *Neurospora* Genome Project [27]. Knockouts that did not germinate and grow in a short time were not further evaluated to determine whether they showed low growth or no growth, so this project did not clearly identify essential genes.

To collect the essential gene sets, we first identified inviable mutants in the e-Compendium, and only retained genes for which we could manually verify their essentiality in the literature. To split the essential genes into a training set and a test set we intersected the list of inviable mutants in the e-Compendium with the list of heterokaryon knockouts from the *Neurospora* Genome Project (http://www.dartmouth.edu/~neurosporagenome/knockouts_completed.html). Genes that were in the intersection became the test set, and the rest remained in the training set.

To collect the non-essential gene sets, we used the homokaryon knockouts from the *Neurospora* Genome Project [82], all of which were experimentally observed to be viable in Vogel's minimal media [102]. A subset of these homokaryons were extensively phenotyped, and these were available at the *Neurospora crassa* Database at the Broad Institute (http://www.broadinstitute.org/annotation/genome/neurospora/Phenotypes.html). This subset became our non-essential training set, while the rest of the homokaryon knockouts became our non-essential test set. Homokaryon knockouts that were in the essential gene set were discarded.

Both the supplemental nutrient rescue training and test sets were initially identified from the e-Compendium and from the book *Neurospora: Contributions of a Model Organism* [1]. They were then confirmed through manual curation of the associated citations. We also used this protocol to identify and confirm known synthetic lethal mutations.

### Comparison of experimental to predicted phenotype data

We simulated Vogel's minimal media (http://www.fgsc.net/methods/vogels.html) [13] with sucrose by including exchange reactions for each metabolite in the media, and limiting sucrose uptake to 1.5 mmol/(gram Dry Weight * hour). Alternative media were formulated *in silico* by adding/removing exchange reactions. Supplemental nutrients were limited to a flux of 3 mmol/(gram Dry Weight * hour).

We simulated gene knockouts by removing reactions that require knocked-out genes. Biomass flux was predicted using limed-FBA, except where stated otherwise. *In silico* growth phenotypes were regarded as viable if the biomass flux exceeded 0.02, and inviable otherwise. FBA was run using the *optimizeCbModel* function from the COBRA Toolbox 2.0.5 [103] using Gurobi 5.0 in Matlab (MathWorks, Natick, MA). MD-FBA was run using Matlab code downloaded from Tomer Shlomi's Research Group's website (http://www.cs.technion.ac.il/~tomersh/methods.html) with Tomlab v7.9 and CPLEX 12.2. This code was modified for our model: all MD-FBA constraints could be satisfied by wild-type on Vogel's minimal media, so we did not need to apply their *addNewExReactions* function. This code allowed 400 seconds per optimization.

### FARM

#### Availability

FARM was originally written in the R language and environment (www.r-project.org). Optimizations utilized the *Rcplex* package in R to call IBM ILOG CPLEX Optimization Studio 12.2 (IBM, Armonk, New York). However, code to reproduce all of our predictions has been made available in MATLAB (MathWorks, Natick, MA). This code is integrated with the COBRA Toolbox 2.0.5 [103]. The MATLAB code is freely available at https://code.google.com/p/fast-automated-recon-metabolism/.

#### limed-FBA

limed-FBA is a linear method which requires a metabolite's concentration to dilute slightly if and only if it is used in active reactions. We model this dilution implicitly by forcing metabolites to compensate for their dilution through *accumulation*. This accumulation can be modeled mathematically using the formalism of FBA, as follows.

Let *S* be a stoichiometric matrix of irreversible reactions (where reversible reactions are represented as two irreversible reactions in opposite directions), *v* be a vector of (non-negative) steady-state metabolic fluxes, and *b* represent a vector of the associated metabolite concentrations changes. Then
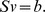
To linearly account for metabolite accumulation, we would like to construct *b* as a linear function of *v* so that *b*_i_ is positive but small when metabolite *i* participates in active reactions, and *b*_i_ is zero otherwise.

To construct such a *b*, we first introduce the binary stoichiometric matrix, *S* ^binary^ [92], whose element at row *i* and column *j* is 1 if and only if the corresponding element of *S≠0*. Now consider *S* ^binary^ *v* for a particular metabolite *i* (this is row *i* of *S* ^binary^ multiplied by *v*). Because *S* ^binary^ and *v* are both non-negative, element *i* of *S* ^binary^ *v* represents the sum of the absolute values of the fluxes that produce and consume metabolite *i*. This quantity is twice the turnover of metabolite *i*. Thus, if metabolite *i* does not participate in active reactions, then element *i* of *S* ^binary^ *v* is zero. Otherwise, if metabolite *i* does participate in active reactions, then element *i* of *S* ^binary^ *v* is positive.

To ensure that the dilution is small when metabolite *i* participates in active reactions, we multiply *S* ^binary^ *v* by ε, where ε is a diagonal matrix whose number of rows and columns is the number of metabolites, and its elements, *ε*_ii_, are small non-negative constants that correspond to each metabolite *i*.

Thus, we set
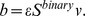
We then implement limed-FBA according to
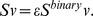
Note that this equation does not hold for all *v*; rather, it only holds for those *v* that are feasible in limed-FBA. This equation can be simplified to
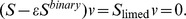
We assign *ε* so that no metabolite's dilution rate can exceed a pre-chosen constant. Here, we set dilution to never exceed 0.1; this choice gives dilution rates on par with those theoretically prescribed by MD-FBA. In general, we assign *ε*_ii_ to be
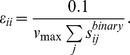
where *v*_max_ is the overall upper bound on the fluxes. We used *v*_max_ = 1000.

Metabolites in biomass already dilute according to FBA. So that we do not double-dilute biomass metabolites, metabolites whose growth-associated dilution is already captured in the pre-defined biomass (e.g. DNA, RNA, protein) are assigned *ε*_ii_ *= 0*.

The use of small numbers such as *ε*_ii_ can create numerical difficulties for optimization solvers, such as IBM ILOG CPLEX. We found that these errors were rare, only occurred when growth was impossible, and disappeared when trying an alternate linear programming solution method (e.g. the simplex method instead of a barrier method). An alternative that we didn't try is to use an exact solver or one with more precision [104].

As a consequence of limed-FBA's linearity, limed-FBA allows more dilution for metabolites with more flux. This is associated with a potential pitfall. Consider a metabolite that is produced as a by-product of growth, but is not connected to an exporter. To maximize growth, this metabolite would need to be depleted. To deplete this metabolite, limed-FBA could potentially “cheat”: it could create a high-flux loop that includes the metabolite, so that the metabolite is allowed to dilute more. If a reaction includes multiple metabolites, then including it in a loop is more likely to have system-wide effects; conversely, reactions with fewer metabolites are less likely to have system-wide effects. In accord with this, we found that limed-FBA used simple transport reactions (e.g. L-glutamine[cytosol]↔L-glutamine[nucleus]) to “cheat.” To address this, we disallow simple transport reactions from contributing towards dilution. We define these simple transport reactions as those that transport metabolites, but involve no chemical change. This disallowance is implemented mathematically by assigning columns in *S* ^binary^ that correspond to these simple transport reactions to be all zeroes.

#### CROP

This algorithm is extensively detailed and compared to similar approaches in Text S3.

#### OnePrune

OnePrune's goal is to send flux through as many reactions as possible given an unlimited amount of the available nutrients. Mathematically, it's defined as a linear goal program:
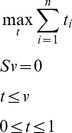
where *S* is a stoichiometric matrix consisting of *n* irreversible reactions. This program does not contain upper bounds on the available nutrient sources, so the only factor limiting reaction flux is connectivity. Thus, in the optimal solution, all *t*_i_'s should be binary: reactions with *t*_i_ of one can carry flux, while the other reactions are blocked.

OnePrune requires an irreversible stoichiometric matrix, but any reversible stoichiometric matrix can be transformed into an irreversible one (by splitting reversible reactions into two irreversible reactions of opposite direction) so this method is completely general. This requirement exists because direct inclusion of a reversible stoichiometric matrix would force OnePrune to deal with absolute values of reaction flux in a manner not amenable to linear programming [105]. Moreover, only an irreversible stoichiometric matrix allows OnePrune to distinguish between different directions of the same reaction.

The set of blocked reactions of an irreversible stoichiometric matrix may differ slightly from those of a reversible stoichiometric matrix. For example, consider a reversible reaction between two metabolites disconnected from the metabolic network: A↔B. In an irreversible stoichiometric matrix, this would be modeled as two reactions: A→B and A ← B. Thus, the reversible reaction is blocked, whereas the two irreversible reactions could carry flux as part of a flux cycle according to FBA. To address this, we implemented OnePrune according to limed-FBA by using S_limed_ as the stoichiometric matrix. This implementation disallows cycles that lack an input flux, so it disallows the cycle of A→B and A ← B. However, limed-FBA does allow some cycles—namely, those not penalized in S_limed_ and those that have an input flux. Thus, reversible reactions that form such cycles (e.g. A[cytosol]↔A[extracellular]) will not be pruned by OnePrune. However, these reversible reactions can be identified *a priori* and addressed with Flux Variability Analysis [57], [58] or the method of Jerby et al. [51]. Furthermore, reversible reactions that form such cycles are rare, and we found that OnePrune's determination of blocked reactions was identical to that of Flux Variability Analysis in practice.

To apply OnePrune to achieve our final pruned model, we allowed all extracellular metabolites in our model to be treated as nutrients.

### Creation of COBRA-compatible SBML model

We exported the model to the COBRA-compatible subset of the Systems Biology Markup Language (SBML) [106], [107]. All SBML identifiers were based on NeurosporaCyc Frame IDs. To avoid using characters disallowed in SBML identifiers, we implemented a substitution scheme. We substituted disallowed characters with their ASCII equivalent, demarcated on both sides by two underscores. For example, “[” has ASCII value 91, so it was substituted by “__91__”. Identifiers beginning with a number were given a prefix of underscore; e.g. “1diacyl” became “_1diacyl”. So that our SBML file can be reversed-transformed into its original character encoding, we wrote an extension to the COBRA toolbox in Matlab. This extension is available at http://code.google.com/p/fast-automated-recon-metabolism. SBML Notes fields contain COBRA-compliant gene associations, pathways, EC numbers, Pubmed IDs, chemical formulae, charge, and NeurosporaCyc and KEGG identifiers [107]. SBML Annotation fields contain MIRIAM-compliant links to InChI identifiers [108], [109].

### Model availability

The metabolic model (Dataset S1) has been deposited at the BioModels Database [110] with accession MODEL1212060001, and is available on the web at http://neurosporacyc.broadinstitute.org.

## Supporting Information

Dataset S1 iJDZ836 SBML.
(XML)

Figure S1 The mechanistic explanation for the *Δarg-4Δarg-11* synthetic lethal prediction is that two alternate routes exist for generating L-ornithine. (A) When *arg-4* is blocked, L-ornithine can be generated from *arg-11*. (B) When *arg-11* is blocked, L-ornithine can be generated via *arg-4*.
(EPS)

Figure S2 The mechanistic explanation for the *ΔsucΔcomplex I* synthetic lethal prediction is that two alternate routes exist in the mitochondrion for regenerating NAD+ from NADH. (A) The first route utilizes the ubiquinone:NADH oxidoreductase enzyme encoded by *complex I*. (B) When ubiquinone:NADH oxidoreductase activity is blocked, NAD+ still can be regenerated by driving the malate dehydrogenase reaction backwards via the pyruvate carboxylase activity encoded by *suc*. When both routes are blocked, the mitochondria unable to regenerate NAD+ from NADH and the resulting double mutant is inviable.
(EPS)

Figure S3 Zoomable metabolic map of *Neurospora crassa*.
(EPS)

Figure S4 Plots glucose uptake rate (in millimoles per gram dry weight per hour) against doubling time (in hours). The black curve depicts the model's predictions using FBA, where doubling time is calculated as ln(2)/growth rate [113], and the blue diamonds depict data points extracted from the experimental literature.
(EPS)

Figure S5 Simulating the effect of oxygen limitation on xylose fermentation. In the first step, xylose is reduced to xylitol via a xylose reductase that has a strong cofactor preference for for NADPH (XR-NADPH) and a weaker preference for NADH (XR-NADH) [114]. In the second step, xylitol is either excreted into the medium or oxidized to xylulose via a NAD+-dependent xylitol dehydrogenase (XDH) [115]. Xylulose is then phosphorylated and enters the pentose phosphate pathway, followed by glycolysis to pyruvate, and is either excreted as ethanol or enters the mitochondria to be oxidized via the TCA cycle [29]. (A) Under anaerobic conditions, our model predicts that XR-NADH is the only source of NAD+ for XDH, so the excess xylitol generated by XR-NADPH is exported, resulting in lower ethanol yields. (B) Under fully aerobic conditions, our model predicts that XDH no longer acts as a bottleneck because NAD+ is regenerated by oxygen via the glycerol phosphate shuttle. Furthermore, enough oxygen is present to generate the NAD+ required by the TCA cycle, which converts all the pyruvate to CO2 and water, resulting in no ethanol production. (C) This figure shows the critical oxygen level where just enough NAD+ is regenerated via the glycerol phosphate shuttle to allow XDH to catalyze all the xylitol, but there is no NAD+ left over to power the TCA cycle, resulting in optimal ethanol yield and productivity. All three predictions of xylitol and ethanol yields are consistent with experimental observation [29].
(EPS)

Table S1 Describes our evidence source for our growth/no-growth annotation of mutants, nutrient rescues, and synthetic lethals.
(XLSX)

Table S2 Comprehensive set of our predicted essential genes, supplemental rescues, and synthetic lethal gene pairs.
(XLS)

Text S1 Describes the 19 inconsistencies between the test set of non-essential genes and the essential gene predictions.
(DOCX)

Text S2 Compares the steps we completed to reconstruct this model to those in the Palsson lab's 96 step protocol [38].
(DOCX)

Text S3 Details the CROP algorithm and compares it to GrowMatch and the Model SEED. This has three sections: 1. objective function, 2. resolving incorrect predictions by restoring growth, and 3. resolving incorrect predictions by suppressing growth.
(DOCX)

Text S4 Simulating the effect of oxygen limitation on xylose fermentation.
(DOCX)
