# Supplementary material for: Reconstruction and Validation of a Genome-Scale Metabolic Model for the Filamentous Fungus Neurospora crassa Using FARM
Source: PLoS Comput Biol. 2013 Jul 18;9(7):e1003126. doi: 10.1371/journal.pcbi.1003126 (PMC3730674; doi:10.1371/journal.pcbi.1003126)
Supplement: Text S1 — Describes the 19 inconsistencies between the test set of non-essential genes and the essential gene predictions. (DOCX) [file pcbi.1003126.s009.docx]

**Test set non-essential inconsistencies**

We identified 19 inconsistencies in the test set where the model predicted inviability and experimental data [1] indicated viability. For some of these inconsistencies, either the predicted essential function was not required for growth, or an unknown gene can perform the same function. For example, five knockouts, Δ*rg-1*, Δ*glp-1*, Δ*NCU06348* (myo-inositol-1-monophosphotase), Δ*chi-1*, and Δ*do*, were predicted to lack essential cell wall components. Two knockouts, Δ*mtr* and Δ*mic-18*, were predicted to lack essential mitochondrial transport of glutamine and 2-oxoglutarate, respectively. The Δ*lys-7* knockout was predicted to lack part of the essential lysine biosynthesis pathway [2].

In one case, a candidate isozyme appears likely. The model predicted that *ndk-1* encodes nucleoside diphosphate kinase activity. However, 10-15% of this activity was observed in the Δ*ndk-1* knockout [3]. Interestingly, nucleoside diphosphate kinase activity was also shown to be performed by adenylate kinase in *E. coli* [4]. Since adenylate kinase in Neurospora is encoded by *adk-1*, this gene may encode a moonlight activity that can account for our incorrect prediction.

Four of the knockout mutants, Δ*NCU08925,* Δ*pan-3,* Δ*mic-30* and Δ*pab-1,* were incorrectly predicted to be essential according to limed-FBA, but correctly predicted to be nonessential by FBA. The reasons for these inconsistencies were described in the main text after the discussion of Figure 4B.

For the remaining five inconsistencies the problem may be with the test data, because, like Δ*pab-1*, other experimental observations of these knockout mutants are consistent with our prediction of essentiality. *his-1* was reported to be a histidine-requiring mutant [5]. *trp-4* was reported to be an L-tryptophan-requiring mutant [6,7] because it was shown to lack anthranilate phosphoribosyltransferase activity [8]. *pyr-2* was reported to be a uracil-requiring mutant [9]. *pyr-1* was reported to be a uridine-requiring mutant [9] because it was shown to lack dihydroorotate dehydrogenase activity [10].

References

1. Colot H, Park G, Turner G, Ringelberg C, Crew C, et al. (2006) A high-throughput gene knockout procedure for Neurospora reveals functions for multiple transcription factors. Proceedings of the National Academy of Sciences 103: 10352-10357.

2. Trupin JS, Broquist H (1965) Saccharopine, an Intermediate of the Aminoadipic Acid Pathway of Lysine Biosynthesis. Journal of Biological Chemistry 240: 2524-2530.

3. Lee B, Yoshida Y, Hasunuma K (2006) Photomorphogenetic characteristics are severely affected in nucleoside diphosphate kinase-1 (ndk-1)-disrupted mutants in Neurospora crassa. Mol Genet Genomics 275: 9-17.

4. Lu Q, Inouye M (1996) Adenylate kinase complements nucleoside diphosphate kinase deficiency in nucleotide metabolism. Proc Natl Acad Sci U S A 93: 5720-5725.

5. Ames BN (1957) The biosynthesis of histidine; D-erythro-imidazoleglycerol phosphate dehydrase. J Biol Chem 228: 131-143.

6. Lester G (1963) Regulation of early reactions in the biosynthesis of tryptophan in Neurospora crassa. J Bacteriol 85: 468-475.

7. Catcheside DG (1960) Complementation among histidine mutants of Neurospora crassa. Proc R Soc Lond B Biol Sci 153: 179-194.

8. Wegman J, DeMoss JA (1965) The enzymatic conversion of anthranilate to indolylglycerol phosphate in Neurospora crassa. J Biol Chem 240: 3781-3788.

9. Caroline DF (1969) Pyrimidine synthesis in Neurospora crassa: gene-enzyme relationships. J Bacteriol 100: 1371-1377.

10. Miller RW (1978) Dihydroorotate dehydrogenase (Neurospora). Methods Enzymol 51: 63-69.
