# Supplementary material for: Reconstruction and Validation of a Genome-Scale Metabolic Model for the Filamentous Fungus Neurospora crassa Using FARM
Source: PLoS Comput Biol. 2013 Jul 18;9(7):e1003126. doi: 10.1371/journal.pcbi.1003126 (PMC3730674; doi:10.1371/journal.pcbi.1003126)
Supplement: Text S2 — Compares the steps we completed to reconstruct this model to those in the Palsson lab's 96 step protocol [38]. (DOCX) [file pcbi.1003126.s010.docx]

**Reconstruction protocol**

Here, we compare our reconstruction to the protocol from Thiele and Palsson [[1](#_ENREF_1)].

***Stage 1: Creating a draft reconstruction***

*1 Obtain genome annotation*

During the reconstruction process, the Neurospora crassa genome sequence has undergone numerous genome assemblies and gene sets. We mapped all genes in the model to the most recent release at the time of submission (assembly 10 gene set v5).

*2 Identify candidate metabolic functions*

Enzyme commission (EC) numbers were inferred from protein sequence using *EFICAz2* [[2-5](#_ENREF_2)].

*3* *Obtain candidate metabolic reactions for these functions*

Reactions associated with EC number predictions and reactions inferred from pathway predictions were imported from MetaCyc using Pathologic [[6-9](#_ENREF_6)].

*4* *Assemble draft reconstruction*

Draft reconstruction was assembled into NeurosporaCyc Pathway/Genome Database (PDGB).

*5* *Collect experimental data*

Experimental evidence for enzyme activities was curated from the Neurospora Community Annotation Project [[10](#_ENREF_10)].

***Stage 2: Manual reconstruction refinement***

*6* *Determine and verify substrate and cofactor usage*

Substrates and cofactors for reactions were determined as follows:

- non-generic reactions were imported directly from MetaCyc into NeurosporaCyc
- generic EC numbers were instantiated by generating all instances of generic substrates and filtering out unbalanced reactions using an extension of the code described in [[9](#_ENREF_9)].
- polymerization reactions were lumped into a single reaction that summarizes the stoichiometry of n iterations of polymerization, where n was arbitrarily set to 8.

*7 Obtain a neutral formula for each metabolite in the reaction*

Neutral formulas were obtained using MarvinBeans major microspecies at pH 7.0

*8 Determine the charged formula for each metabolite in the reaction.*

Charged formulas were obtained using MarvinBeans major microspecies at pH 7.2 [[11](#_ENREF_11)] for the cytoplasm, and pH 6.8 for the vacuole.

*9 Calculate reaction stoichiometry.*

Reaction stoichiometries for charged formulae were calculated using the Pathway-tools reaction balancer code [[9](#_ENREF_9)].

*10 Determine reaction directionality*

Reaction directionality was computed from the Gibbs free energy using the group contribution method [[12](#_ENREF_12)].

*11 Add information for gene and reaction localization*

Gene locations were obtained from the Neurospora Community annotation project gene ontology terms [[10](#_ENREF_10)]. Reaction locations were curated from the literature [[13](#_ENREF_13)].

*12 Add subsystem information to the reaction*

Pathways (subsystems) were computed using Pathologic [[6](#_ENREF_6)].

*13 Verify GPR association*

Gene-reaction associations were computed using EFICAz, followed by enzymes identified by the Pathway hole filling algorithm [[2](#_ENREF_2),[7](#_ENREF_7)]. Enzyme complexes were semi-automatically constructed using the Pathway-tools protein-complex building tool followed by manual curation [[6](#_ENREF_6)].

*14 Add metabolite identifier*

SMILES and InChI’s were computed from the chemical structure of the charged compounds in NeurosporaCyc. External references to equivalent KEGG, Pubchem, and ChEBI identifiers were also exported, when available.

*15 Determine and add the confidence score*

Confidence scores per reaction were based on the following types of evidence:

a EFICAz precision probability

b Is enzyme in a curated pathway?

c Does enzyme have experimental evidence from literature citations?

d Is reaction predicted to be in NeurosporaCyc by Pathologic based on pathway holes?

*16 Flag those reactions for which information from other organisms was used*

Enzyme functions inferred using sequence homology were marked as such using the Pathway-tools evidence ontology [[14](#_ENREF_14)]

*17 Add references and notes based on experimental information*

For enzyme functions supported by literature citations, we also included an evidence code using the Pathway-tools evidence ontology [[14](#_ENREF_14)]

*18 Repeat Steps 6–17 for all those genes that were identified in the draft reconstruction*

*19 Add spontaneous reactions to the reconstruction*

Spontaneous reactions were added from MetaCyc.

*20 Add extracellular and periplasmic transport reactions to the reconstruction*

Transport reactions were based on manual curation of the automated transport predictions [[8](#_ENREF_8)]. Extracellular reactions were based on evidence from literature citations.

*21 Add exchange reactions to the reconstruction*

Exchange reactions were added for all nutrients in the media.

*22 Add intracellular transport reactions to the reconstruction*

Intracellular transport reactions were added based on evidence from literature citations. Cell components were categorized according to Pathway-tools extension of the Cell Component Ontology (http://bioinformatics.ai.sri.com/CCO) [[15](#_ENREF_15)].

*23 Draw metabolic map*

An initial draft of the metabolic map was automatically generated using the NeurosporaCyc cellular overview [[9](#_ENREF_9)]. Further work on the map was done in Adobe Illustrator.

*24 Determine the chemical composition of the cell, i.e., protein, RNA, DNA, lipids, and cofactor content*

Biomass was decomposed into separate synthesis reactions for DNA, RNA, amino acids, carbohydrates, lipids, sterols, essential cofactors, growth-associated ATP maintenance, and secondary metabolites. This decomposition allows for modular construction of biomass objective functions depending on the goals of the FBA. For example, to predict wild-type fluxes we compiled all of these elements into full biomass composition. Wild-type biomass contains substantial secondary metabolites, such as carotenoids (which give Neurospora its color), sphingolipids, and ergosterol, but mutants that do not contain these do (slowly) grow. Consequently, to predict gene essentiality we removed these secondary metabolites to form essential biomass composition.

*25 Determine the amino acid content either experimentally (option A) or by estimation (option B)*

Amino acid composition was estimated from parsing Neurospora Uniprot's amino acid FASTA file (option B).

*26 The molar percentage and molecular weight of each amino acid must be used to calculate the weight per mol protein*

For each amino acid, the total number of times each amino acid appears in the Uniprot file was computed. This number was divided by the total number of amino acids to compute the Mol fraction (mol AA/ mol protein). This number was multiplied by the molecular weight (g AA/mol AA) of the amino acid to derive the g AA/mol protein. We then divide the Mole fraction by g AA/mol protein *1000 to obtain the mmol AA/g protein. This is used as the stoichiometric coefficient.

*27 Determine the nucleotide content either experimentally (option A) or by estimation (option B)*

DNA content was estimated from the number of A/C/G/T in the genome (including mitochondrial and chromosomal). RNA content was estimated from the Broad Institute Neurospora “transcripts” file. Expression for each gene was assumed to be equal.

*28 Calculate the fractional distribution of each nucleotide to the biomass composition by repeating Step 26*

*29 Determine the lipid content*

Lipid content was divided into glycerophospholipid composition and triacylglyceride (TAG) composition. Glycerophospholipid composition is composed of an L-1-phosphatidyl-ethanolamine, a phosphatidylcholine, an L-1-phosphatidylserine, and an L-1-phosphatidyl-inositol. The fatty acid stoichiometric composition of phospholipids on minimal media were derived from figure 1C of [[16](#_ENREF_16)]. The headgroup stoichiometric composition was derived from table 6.4 of [[13](#_ENREF_13)]. Since there are two acyl groups per phosphoglycerol, we double fatty acid composition (which adds up to 1) to get the correct stoichiometry. Fatty acid stoichiometric coefficients for TAGs under minimal medium were derived from Figure 1C in [[16](#_ENREF_16)]. Since there are three acyl groups per triacylglycerol, we tripled the fatty-acid composition (which added up to 1).

*30 Determine the content of the soluble pool*

The soluble pool of metabolites such as NAD, thioredoxin, ferredoxin, and glutathione was not added to the biomass, because limed-FBA already requires the biosynthesis of cofactors and vitamins required for growth. However, we found that even with limed-FBA we needed to include the reaction FADH2 → FAD to recapitulate gene essentiality data.

*31 Determine the ion content*

Ion content was not determined for this release of the model.

*32 Determine growth-associated maintenance (GAM) stoichiometric ratio*

We identified a GAM ratio that provided the best fit to experimentally observed growth rates on Vogel’s minimal media with glucose.

*33 Compile and add biomass reaction to the reconstruction*

See step 24 above.

*34 Add non-growth-associated maintenance (NGAM) requirements of the cell to maintain turgor pressure and other non-metabolic cellular processes*

The lower bound for this reaction was taken from *A. niger*, where it was estimated from growth experiments in continuous culture [[17](#_ENREF_17)].

*35 Add demand reactions to the reconstruction*

SecondaryMetaboliteComposition contains non-essential compounds known to be produced by Neurospora.

*36 Add sink reactions to the reconstruction*

Our final model does not contain sink reactions. To deal with protein substrates of reactions, such as thioredoxin, we created reversible reactions of the form amino acids ↔ thioredoxin.

*37 Determine growth medium requirements*

Vogel’s Medium N is used almost universally for vegetative growth and stock culture [[13](#_ENREF_13)]. Although citrate is in the nutrient media, citrate is not permeable to Neurospora, so it cannot serve as an exchange metabolite.

***Stage 3: Conversion from reconstruction to mathematical model***

Our reconstruction was stored in the NeurosporaCyc Pathway/Genome database, which interacts with the Pathway-tools software [[18](#_ENREF_18)]. Pathway-tools is written in the LISP programming language. Thus, we wrote custom tools in LISP to convert to a mathematical model. This conversion instantiated NeurosporaCyc's generic reactions, and transferred NeurosporaCyc into text files. These files contained the stoichiometric matrix; notes and additional evidence about each reaction; metabolites and external references to database independent and dependent identifiers; gene protein reaction associations; reactions that were flagged, with a reason for the flag; nutrient media; pathways for the reactions; and the subset of reactions (with direction) that defined the model. Finally, to communicate our model to the systems biology community, we used python to generate an SBML model from these files.

*38 Load nc10.xml reconstruction into Matlab*

*39 Verify S matrix*

*40 Set objective function*

*41 Set objective function*

*42 Set simulation constraints*

We then validated the SBML in Matlab, using the Cobra Toolbox. All code has been made available at: http://code.google.com/p/fast-automated-recon-metabolism.

***Stage 4: Network evaluation = ‘Debugging mode’***

*43 Test if the network is mass and charge balanced. Check for stoichiometrically unbalanced reactions*

*44 Evaluate stoichiometrically unbalanced reactions*

*45 Identify metabolic dead ends*

*46 Identify candidate reactions to fill gaps*

*47 Add gap reactions to the reconstruction*

*48 Add notes and references to dead-end metabolites*

*49 Add missing exchange reactions to model*

*50 Set exchange constraints for a simulation condition*

Network debugging was performed during the phenotype-directed curation phase of our model reconstruction process. Gaps, missing exchange reactions, and candidate reactions to fill the gaps were all identified by FARM.

***Test for stoichiometrically balanced cycles or Type III pathways***

*51 Test for Type III pathways*

*52 Analyze the output if Type III pathways are found*

*53 Identify Type III pathways*

*54 Analyze directionality of each reaction participating in a Type III pathway*

*55 Analyze if any reaction participating in a Type III pathway may be falsely included in the reconstruction by reviewing the supporting evidence*

*56 If none of the reactions or reaction directions can be corrected based on experimental or thermodynamic information, you can try to iteratively limit the directionality of the loop reactions*

*57 Adjust the directionality for all those reactions identified in Steps 54–56, note the change and reasons*

*58 After eliminating a reaction direction or a deletion of a reaction, repeat the Type III pathway analysis*

*59 Recompute gap list*

limed-FBA disallows flux cycles that lack an input flux, so we this eliminates most type III pathways, so we did not need to manually adjust the reaction directionality of the model to prevent these cycles from occuring.

***Test if biomass precursors can be produced in standard medium***

*60 Obtain the list of biomass components*

*61 Add demand function for each biomass precursor*

*62 For each biomass component, perform the following test: Change objective function to the demand function*

*63 Maximize ('max') for new objective function*

*64 Identify reactions that are mainly responsible for synthesizing the biomass component*

*65 For each of these reactions, follow the wire diagram given in Figure 14*

*Steps 60-65 were tested using dung-FBA, which is a goal programming approach discussed in the CROP Supplement. dung-FBA allowed us to test for all biomass components simultaneously, and see which ones could be produced and which could not*

*66 Test if biomass precursors can be produced in other growth media*

We tested that growth could be achieved in other media known to support Neurospora's growth, such as using acetate as a sole carbon source.

***Test if the model can produce known secretion products***

*67 Collect a list of known secretion products and medium conditions.*

*No secretion products were found*

*68 Set the constraints to the desired medium condition*

*69 Change the objective function to the exchange reaction of your secretion product:*

*70 Maximize ('max') for the new objective function*

Secretion products for Neurospora are not known.

***Test if the model can produce a certain ratio of two secretion products***

*71 Set the constraints to the desired medium condition*

*72 Verify that both by-products can be produced independently*

*73 Add a row to the S matrix to couple the by-product secretion reactions*

*74 Change the objective function to the exchange reaction of one of your secretion products*

*75 Maximize for the new objective function*

Secretion products for Neurospora are not known.

***Check for blocked reactions***

*76 Change simulation conditions to rich medium*

*77 Run analysis for blocked reactions*

*78 Connect reaction to remaining network*

Blocked reactions were identified and removed using OnePrune.

***Compute single-gene deletion phenotypes***

*79 Compute single-gene deletion phenotypes*

*80 Compare with experimental data*

We used CROP to compare our single-gene deletion phenotype predictions with experimental data to improve the model.

***Test for known incapabilities of the organism***

*81 Set simulation condition*

*82 Use single-reaction deletion to identify candidate reactions that enable the model's capability despite known incapability*

We used CROP to improve our model using known incapabilities of the organism under different nutrient conditions.

***Test if the model can predict the correct growth rate or other quantitative properties***

*83 Compare the predicted physiological properties with the known properties*

We do not know Neuropora's nutrient uptake rates, so we cannot accurately gauge its growth rate given uptake. However, our predicted growth rate is consistent with observed growth rate in Vogel’s medium, when we limit sucrose uptake to 5 mmol/(g * hr).

***Test if the model can grow fast enough***

*84 Optimize for biomass in different medium conditions and compare with experimental data*

*85 Test if any of the medium components are growth limiting.*

*86 Maximize for biomass*

*87 Determine the reduced cost associated with network reactions when optimizing for objective function*

We were unable to obtain nutrient uptake rates in order to determine if our growth rate was fast enough.

***Test if the model grows too fast***

*88 Optimize for biomass reaction in different medium conditions and compare with experimental data*

*89 Verify that the model constraints are set as intended*

We were unable to obtain nutrient uptake rates in order to determine if our growth rate was too fast.

***Carry out one or more of the following tests to identify possible errors in the network***

*90 Verify that all fractions and precursors in the biomass reaction are consistent with the present knowledge*

*91 Identify shuttling reactions*

*92 Re-investigate the thermodynamic information associated with the network reaction*

*93 Use single-reaction deletion to identify single reactions that may enable the model to grow too fast*

*94 Reduced cost*

We verified that all fractions and precursors of biomass are consistent with the literature. Citations for each biomass precursor are listed on the associated NeurosporaCyc web pages.

***Data assembly and dissemination***

*95 Print Matlab model content*

*96 Add gap information to the reconstruction output*

In addition to our SBML, associated information is available from our structured database NeurosporaCyc, at http://neurosporacyc.broadinstitute.org.

References

1. Thiele I, Palsson B (2010) A protocol for generating a high-quality genome-scale metabolic reconstruction. Nature Protocols 5: 93-121.

2. Arakaki A, Huang Y, Skolnick J (2009) EFICAz2: enzyme function inference by a combined approach enhanced by machine learning. BMC Bioinformatics 10: 107.

3. Arakaki A, Tian W, Skolnick J (2006) High precision multi-genome scale reannotation of enzyme function by EFICAz. BMC Genomics 7: 315.

4. Tian W, Skolnick J (2003) How Well is Enzyme Function Conserved as a Function of Pairwise Sequence Identity? Journal of Molecular Biology 333: 863-882.

5. Tian W, Arakaki A, Skolnick J (2004) EFICAz: a comprehensive approach for accurate genome-scale enzyme function inference. Nucleic Acids Research 32: 6226.

6. Karp P, Paley S, Romero P (2002) The Pathway Tools software. Bioinformatics (Oxford, England) 18 Suppl 1: S225-S232.

7. Green M, Karp P (2004) A Bayesian method for identifying missing enzymes in predicted metabolic pathway databases. BMC Bioinformatics 5: 76.

8. Lee T, Paulsen I, Karp P (2008) Annotation-based inference of transporter function. Bioinformatics 24: i259-i267.

9. Latendresse M, Krummenacker M, Trupp M, Karp P (2012) Construction and completion of flux balance models from pathway databases. Bioinformatics 28: 388-396.

10. Dunlap J, Borkovich K, Henn M, Turner G, Sachs M, et al. (2007) Enabling a community to dissect an organism: overview of the Neurospora functional genomics project. Adv Genet 57: 49-96.

11. Legerton TL, Kanamori K, Weiss RL, Roberts JD (1983) Measurements of cytoplasmic and vacuolar pH in Neurospora using nitrogen-15 nuclear magnetic resonance spectroscopy. Biochemistry 22: 899-903.

12. Jankowski M, Henry C, Broadbelt L, Hatzimanikatis V (2008) Group Contribution Method for Thermodynamic Analysis of Complex Metabolic Networks. Biophysical Journal 95: 1487-1499.

13. Davis R (2000) Neurospora contributions of a model organism. New York, NY: Oxford University Press.

14. Karp PD, Paley S, Krieger CJ, Zhang P (2004) An evidence ontology for use in pathway/genome databases. Pacific Symposium on Biocomputing Pacific Symposium on Biocomputing: 190-201.

15. Ashburner M, Ball CA, Blake JA, Botstein D, Butler H, et al. (2000) Gene ontology: tool for the unification of biology. The Gene Ontology Consortium. Nature Genetics 25: 25-29.

16. McKeon TA, Goodrich-Tanrikulu M, Lin JT, Stafford A (1997) Pathways for fatty acid elongation and desaturation in Neurospora crassa. Lipids 32: 1-5.

17. Andersen M, Nielsen M, Nielsen J (2008) Metabolic model integration of the bibliome, genome, metabolome and reactome of Aspergillus niger. Molecular Systems Biology 4.

18. Karp P, Paley S, Krummenacker M, Latendresse M, Dale J, et al. (2010) Pathway Tools version 13.0: integrated software for pathway/genome informatics and systems biology. Brief Bioinform 11: 40-79.
