# Supplementary material for: Reconstruction and Validation of a Genome-Scale Metabolic Model for the Filamentous Fungus Neurospora crassa Using FARM
Source: PLoS Comput Biol. 2013 Jul 18;9(7):e1003126. doi: 10.1371/journal.pcbi.1003126 (PMC3730674; doi:10.1371/journal.pcbi.1003126)
Supplement: Text S3 — Details the CROP algorithm and compares it to GrowMatch and the Model SEED. This has three sections: 1. objective function, 2. resolving incorrect predictions by restoring growth, and 3. resolving incorrect predictions by suppressing growth. (DOCX) [file pcbi.1003126.s011.docx]

**CROP**

Here, we describe the Consistent Reproduction Of growth/no-growth Phenotype (CROP) algorithm of FARM. This optimization-based algorithm reconciles experimental with predicted growth phenotypes by suggesting reactions additions/deletions to the metabolic network.

Our exposition is split into three sections. (1) We describe the novel objective function that CROP uses to incorporate diverse evidence. We then describe our complete solution in the two situations where CROP is needed: (2) when no-growth is predicted, but growth is experimentally observed; and (3) when growth is predicted, but no growth is experimentally observed.

1. Objective function

CROP maximizes an objective function that has weights associated with each reaction. These reactions are represented as being irreversible, where reversible reactions are split into two irreversible reactions, so that CROP can also choose among the reaction directions.

We derive the weights using maximum a posteriori (MAP) estimation. This estimation requires the probabilities that a reaction is biochemically and thermodynamically feasible, because CROP’s purpose is to identify a stoichiometric matrix that defines the feasible space of reaction fluxes.

## Probability of biochemical feasibility

A reaction is biochemically feasible if it can occur. Spontaneous reactions are always biochemically feasible. Transport reactions that require a transporter are biochemically feasible if and only if the genome encodes the transport function. Non-spontaneous enzyme-catalyzed reactions are biochemically feasible if and only if the genome encodes the enzyme function. The probability that a non-spontaneous reaction is biochemically feasible depends on the probability that the genome encodes the required function.

Transport reactions were assigned a probability of one if they had associated evidence in NeurosporaCyc; otherwise they were assigned a probability of zero. Non-spontaneous enzyme-catalyzed reactions were assigned a probability from the enzyme function predictor EFICAz [[1](#_ENREF_1)]. When there was additional evidence available in NeurosporaCyc, such as genome annotation, pathway prediction, and experimental evidence from the literature, this evidence was used to update the assigned probabilities.

## Probability of thermodynamic feasibility

A reaction (in a given direction) is thermodynamically feasible if and only if its Gibbs free energy *ΔG<0*. *ΔG* is a function of the Gibbs free energy at standard state *ΔG^0^* and of the substrate/product concentrations. This function is *ΔG=* *ΔG^0^+RTln(Q)*, where *R* is the gas constant, *T* is the temperature in degrees Kelvin, and *Q* is the mass-action ratio. For a biochemical equation of the form *aA + bB →cC + dD*, we have:

$\Delta G'=\Delta G'^{0}+RTln\left(\frac{[C]^{c}[D]^{d}}{[A]^{a}[B]^{b}}\right)$ .

We can estimate *ΔG* from estimates of *ΔG^0^* [[2](#_ENREF_2)] and from physiological ranges of metabolite concentrations. We can further assess the variance of *ΔG* from the variance of *ΔG^0^*. Taken together, we can use the approach of Fleming et al. [[3](#_ENREF_3)] to estimate the *probability* that a given reaction direction is thermodynamically feasible.

Yet, when using this approach, we found that thermodynamically infeasible reactions were not sufficiently penalized, so we developed a novel way to estimate the probability that a given reaction direction is thermodynamically feasible. The reason that thermodynamically infeasible reactions were not sufficiently penalized was because metabolite concentrations were allowed to freely vary within the physiological range. We could not address this problem directly, because we do not have comprehensive measurements of metabolite concentrations across conditions. We do not even have comprehensive measurements of concentrations of cofactors, such as ATP, across conditions. This situation applies to all organisms, not only Neurospora [[4](#_ENREF_4)].

However, metabolite concentrations enter into as ratios, not as absolutes. And these ratios -- such as NAD+/NADH, NADP+/NADPH, and ATP/ADP -- are bounded above and below by thermodynamics [[5](#_ENREF_5)]. Thus, we used known bounds on the ratios of currency metabolites to bring our thermodynamic probability estimates more in line with known thermodynamic feasibility. To illustrate, consider the reaction: glyoxylate + NADPH + H^+^ 🡪 glycolate + NADP^+^, with ΔG^0^ estimate -7.68 kcal/mol. We have:

$\Delta G'=-7.68+RTln\left(\frac{[glyoxylate][NADPH][H^{+}]}{[glycolate][NADP^{+}]}\right)$.

The maximum thermodynamic ratio of [NADP]/[NADPH] has been estimated to be 1.1 [[2](#_ENREF_2)], and it is thought that organisms try to maintain this ratio near its maximum. Thus, we assumed that in the cytosol [NADP]/[NADPH]=1.1. To estimate *ΔG* we also assumed a neutral pH (i.e. [H^+^]=10^-7^).

$$\begin{matrix} \Delta G'=-7.68+RTln\left(\frac{10^{-7}[glyoxylate]}{1.1[glycolate]}\right) \\ \Delta G'=-7.68-16RTln\left(\frac{[glyoxylate]}{[glycolate]}\right) \end{matrix}$$

Now, we can use the physiological range of metabolite concentrations to constrain [glyoxylate] and [glycolate]. But since we’ve already used the maximum thermodynamic ratio of [NADP]/[NADPH], our confidence interval for $\Delta G'$ is much tighter, so the probability estimate is much more reliable. To our knowledge, using thermodynamic bounds on metabolite concentration ratios to obtain more accurate probabilistic estimates of thermodynamic feasibility is novel.

## Maximum a posteriori (MAP) estimation

Our derivation of weights associated with each reaction is motivated by MAP estimation. The reactions are irreversible reactions from a metabolic database, such as MetaCyc. We begin by defining a binary variable *Z*_i_ for each reaction *i* that denotes whether it is physiologically feasible for Neurospora to carry flux through reaction *i* in a condition:

$Z_{i}=\left\{\begin{matrix} 1 \text{reaction }i\text{ is physiologically} \text{feasible} \\ 0 \text{otherwise} \end{matrix}\right.$.

*Z*_i_=1 corresponds to the event that reaction *i* belongs in the “true” metabolic network. Thus, CROP’s purpose is to decide which *Z*_i_’s should be one and which should be zero; this decision specifies the metabolic network.

We relate our data on probabilities of biochemical and thermodynamic feasibility (described above) to probabilities of physiological feasibility.

$$\begin{matrix} p_{i}=P(\text{reaction }i\text{ is physiologically} \text{feasible)} \\ p_{i}=P(\text{reaction }i\text{ is biochemically and thermodynamically feasible)} \\ p_{i}=P(\text{reaction }i\text{ is biochemically feasible)}P\text{(reaction }i\text{ is thermodynamically feasible)} \end{matrix}$$

We then use our data *p*_i_ to formulate the MAP estimate for the vector of *Z*_i_’s. This vector estimate is denoted by . This gives:

By definition of p_i_, we know that:

$$P(Z_{i}=1|p_{i})=p_{i}$$

and similarly,

$P(Z_{i}=0|p_{i})=1-p_{i}$ .

Now, we represent $P(Z_{i}|p_{i})$ as a mixture model, which “mixes” the cases where *Z*_i_=1 and *Z*_i_=0:

$$\begin{matrix} P(Z_{i}|p_{i})=Z_{i}P(Z_{i}=1|p_{i})+(1-Z_{i})P(Z_{i}=0|p_{i}) \\ P(Z_{i}|p_{i})=Z_{i}p_{i}+(1-Z_{i})\left(1-p_{i}\right). \end{matrix}$$

We log-transform to obtain a form that matches ,

.

We would like to optimize , as shown in , with constraints regarding the network properties. (The full optimization is discussed in the sections below.) Because the *Z*_i_ variables are binary, this optimization problem is very computationally demanding – in fact, it is NP-hard. So that this already demanding optimization problem is tractable using available solvers, its objective function must be linear or quadratic, and for speed, it is desirable for it to be linear.

So we sought a linear approximation to . To model this logarithmic function as a linear one, we appeal to the logarithm’s first-order Taylor series approximation around 0: *ln(1+x)≈x* for *-1<x≤1*. The appeal is implemented as:

$\begin{matrix} \ln\left[Z_{i}p_{i}+(1-Z_{i})\left(1-p_{i}\right)\right]=\ln\left[2Z_{i}p_{i}+1-Z_{i}-p_{i}\right] \\ \ln\left[Z_{i}p_{i}+(1-Z_{i})\left(1-p_{i}\right)\right]\approx2Z_{i}p_{i}-Z_{i}-p_{i} \end{matrix}$ .

To exclude the possibility that $Z_{i}p_{i}+(1-Z_{i})\left(1-p_{i}\right)=0$, which would pose a problem for the logarithm, we only need to assume that *0<p<1*.

We now put the above steps together to show our linear approximation for the MAP estimate:

where terms without *Z*_i_ were removed, because they are not involved in the optimization. We simplify by multiplying through by 1/2,

,

which can be stated in matrix notation as:

.

This implies that our objective function is: $Z\left(p-0.5\right)$.

2. Resolving incorrect predictions by restoring growth

In this section, we mathematically deal with conditions where no-growth is predicted, but growth is experimentally observed. (A condition is defined by what genes are knocked-out and what nutrients are available.) Thus, the goal here is to identify model changes that restore growth in a given condition.

There are several model changes that can restore growth. One model change that can restore growth is to assign a knocked-out gene an isozyme, so that the gene knockout does not lead to reaction removals. This change is implemented by modifying the gene-protein-reaction (GPR) associations. Another model change that can restore growth is to remove metabolites from the biomass composition, since this reduces the requirements for growth. This is implemented by changing the biomass composition in the stoichiometric matrix. If neither of these model changes were appropriate, we sought to restore growth by adding reactions to the model from MetaCyc or from NeurosporaCyc reactions that were not in the model. To identify the reactions to add to the model to restore growth, we implemented an optimization.

This optimization can be modeled as a large mixed-integer linear program (MILP). Consider a stoichiometric matrix *S* with irreversible reactions from a model and from a database, a vector of binary variables *z* with associated non-negative *weights* corresponding to reactions in the database, and a vector of reaction upper bounds $U$. For example, consider *U*. If the condition limits the *glucose* uptake reaction to 10, then we set *U*_glucose_=10, and for all other reactions we set *U=*1000, as a loose upper bound. If the condition includes a gene knockout, then for reactions *KO* that require the gene, we set *U*_KO_=0. Now, assume that the *ATP* flux must be at least *atp_maintenance*, and that growth, represented by *v*_biomass_, must be at least *minimal_growth*. Then, the general form of the MILPs is:

$\begin{matrix} \min_{z} weights^{T}z \\ Sv=0 \\ 0\leq v_{i}\leq U_{ i} \text{for} i\in\text{model} \\ 0\leq v_{i}\leq U_{ i}\cdot z_{i} \text{for} i\in\text{database} \\ v_{\text{biomass}}\geq\text{minimal\_growth} \\ v_{\text{ATP}}\geq\text{atp\_maintenance} \\ z\in\{0,1\} \end{matrix}$ .

The main difference between MILPs is how they assign *weights* for reactions from the database. GrowMatch [[6](#_ENREF_6)] treats all reactions as having the same weight, so it minimizes the total number of reactions to add. The Model SEED [[7](#_ENREF_7),[8](#_ENREF_8)] assigns these weights based on diverse evidence. Since there is one binary variable per reaction in the database, these MILPs can be very large, so they are time-consuming to solve.

Our formulation is similar to program . Since program is a minimization, the weights in our objective function (derived in the previous section) would be *0.5-p*. To exploit the structure of program for efficient solving, though, we require that our weights be non-negative. So, we slightly modify our objective function, and instead use: $weights=1-p$, where *0<p<1*.

With non-negative weights, we are able to solve in only a few seconds by first solving a *linear relaxation* of the MILP. This linear relaxation allows all of the variables *z*_i_, which were formerly restricted to be binary, to lie anywhere between 0 and 1, inclusive (i.e. anywhere in [0,1]). Solving this linear relaxation is very fast, since it is a linear program.

The idea behind this linear relaxation is that it minimizes a weighted sum of flux through the database reactions. Thus, reactions not assigned flux in the relaxation are not needed to restore growth; conversely, reactions assigned flux are likely to have a low weight and contribute to restoring growth. Then, the set of reactions whose *z*_i_>0 in the linear relaxation becomes the set of reactions to add to the model, and these reactions restore growth.

This set of reactions returned by the relaxation -- the *relaxation set* -- is not necessarily optimal for . However, we found this set of reactions to be very similar to the optimal set in practice, and furthermore we could efficiently improve the set in two ways. (I) We can treat the *relaxation set* as if it were the set of all database reactions, and then optimize program normally. Because the *relaxation set* is much smaller than the set of all database reactions, this optimization runs remarkably quickly. (II) We can solve program using the *relaxation set* as a high-quality feasible starting point (often called a *warm-start*), which can save the optimizer substantial computational time [[9](#_ENREF_9)].

3. Resolving incorrect predictions by suppressing growth

In this section, we mathematically deal with conditions where growth is predicted, but no growth is experimentally observed. Thus, the goal here is to identify model changes that suppress *in silico* growth in a given condition. Importantly, these model changes should not have the side-effect of suppressing growth in other conditions where growth is experimentally observed, such as the condition of wild-type in minimal medium.

There are several model changes that can suppress growth. These are basically the reverse of those that restore growth. If the knocked-out gene encodes an isozyme, then removing its isozyme, or requiring both gene products as subunits of an enzyme complex, will lead to reaction removal. Reaction removal can suppress growth if the reaction is essential. Another model change that can suppress growth is to add metabolites to the biomass composition, since this increases the requirements for growth. These two model changes can also be implemented together. However, if neither of these model changes were appropriate, we sought to suppress growth by removing reactions from the model. To identify the reactions to remove from the model to suppress growth, we implemented an optimization.

To illustrate the principles involved in the optimization, we first provide a generic optimization-based solution strategy. We then compare the generic strategy to the optimizations of GrowMatch [[6](#_ENREF_6)] and Model SEED [[7](#_ENREF_7),[8](#_ENREF_8)]. GrowMatch, Model SEED, and CROP all solve this problem using a large mixed integer linear program (MILP).

All of these MILPs involve the dual linear program to FBA. The dual linear dual linear program to FBA is required, because it provides an upper bound on growth, and no-growth is characterized by an upper bound on growth of zero. If these programs tried instead to simply set the FBA growth flux to zero, these programs would only represent that no-growth was feasible; they would not represent that growth is infeasible.

## Generic solution strategy

This strategy independently examines each condition where growth is predicted, but no growth is experimentally observed. For each such condition, a bi-level MILP is created. The purpose of this MILP is to find the optimal set of reactions to remove such that *in silico* growth becomes infeasible in this condition, but remains feasible for a set of control conditions, such as wild-type in minimal medium.

This MILP has the same variables as described previously: *S, U, z, weights,* *minimal_growth,* and *atp_maintencance*. However, there are now two conditions: the condition where no growth is experimentally observed, and wild-type in minimal medium. Then, the vector *v* represents flux for the condition where no growth is experimentally observed, and has upper bounds *U*_no-growth_; the vector *w* represents flux for wild-type in minimal medium, and has upper bounds *U*_wt_.

$$\begin{matrix} \min_{z} weights^{T}z \\ \max_{v} v_{\text{biomass}} \\ Sv=0 \text{(inner} \text{problem)} \\ 0\leq v_{i}\leq U_{no-growth, i}\cdot z_{i} \\ v_{\text{biomass}}\leq\text{minimal\_growth} \\ Sw=0 \\ 0\leq w_{i}\leq U_{wt, i}\cdot z_{i} \\ w_{\text{biomass}}\geq\text{minimal\_growth} \\ w_{\text{ATP}}\geq\text{atp\_maintenance} \\ z\in\{0,1\} \end{matrix}$$

The outer minimization problem represents the modeler's optimization to find the optimal set of reactions to remove such that *in silico* growth represented by *v*_biomass_, falls below *minimal_growth*. The constraint *v*_biomass_≤*minimal_growth* implies that when there are no solutions that reduce growth to *minimal_growth*, program will not report on solutions that partially reduce growth. However, such solutions may be helpful in cases where no complete solutions can be identified.

The inner maximization problem of represents FBA, although without the requirement for ATP. We removed this requirement, because it may not be satisfied in the no-growth condition.

Program is a bi-level MILP. To transform it into a single-level MILP, the inner maximization problem must be transformed into a set of constraints. This has been done using an ingenious technique proposed by Maranas’ lab [[10](#_ENREF_10)] based on the strong duality theorem of linear programming. In this technique, the inner problem is transformed into a set of constraints by including the constraints of the inner problem’s dual, and then constraining the objectives of the inner problem and the inner problem’s dual to be equal.

Before illustrating the primal and dual formulations when *z* is included, we first show this without *z* for simplicity. Without *z*, the primal is FBA. To represent FBA in a way that will simplify the derivation of its dual, we represent FBA without unnecessary bounds. So if there is only a single limiting nutrient, e.g. *v*_glucose_*≤10*, then we represent FBA with no other upper bounds. Moreover, *v*_glucose_ does not need an explicit lower bound of zero, because it will never be negative. As with the inner problem above, we also remove the ATP maintenance requirement. Then, the form of the primal LP is:

$$\begin{matrix} \max_{v} c^{T}v=v_{\text{biomass}} \\ Sv=0 \\ 0\leq v_{i} \text{for} i\neq\text{glucose} \\ v_{\text{glucose}}\leq10 \end{matrix}$$

Now, we need the dual. The reason we need the dual is because we want to model an upper bound on growth. To derive the dual, we follow the approach in [[11](#_ENREF_11)], which uses the Lagrangian *L*:

$$\begin{matrix} L=c^{T}v+mSv-\sum_{\text{i}\neq\text{glucose}} r_{i}\cdot v_{i}+r_{\text{glucose}}\cdot(10-v_{\text{glucose}}) \\ L=10r_{\text{glucose}}+(c+S^{T}m-r)v \end{matrix}$$

Then, the Lagrangian dual problem is:

$$\begin{matrix} \min_{m,r} \max_{v} 10\cdot r_{\text{glucose}}+(c+S^{T}m-r)v \\ r_{\text{glucose}}\geq0 \\ r_{\text{i}}\leq0 \text{for} i\neq\text{glucose} \end{matrix}$$

which is equivalent to:

$$\begin{matrix} \min_{m,r} \text{ }10r_{\text{glucose}} \\ S^{T}m-r=-c \\ r_{\text{glucose}}\geq0 \\ r_{i}\leq0 \text{for }i\neq\text{glucose} \end{matrix}$$

where *S^T^* is the transpose of the stoichiometric matrix *S, r* is the dual variable associated with *r*eaction constraints (e.g. *v≥0* and v_glucose_*≤10*), and *m* is the dual variable associated with *m*etabolite constraints (i.e. a row of *Sv=0*).

Now we address the case where there is a binary decision variable per reaction, *z*. To include *z* in the optimization, we need a loose upper bound, *U. U* is required to be sufficiently large such that the constraint *v*_i_$\leq$*U*_i_*z*_i_ does not affect the optimum when *z*_i_*=1*. Then, the primal becomes:

$$\begin{matrix} \max_{v} c^{T}v=v_{\text{biomass}} \\ Sv=0 \\ 0\leq v_{i}\leq U_{i}\cdot z_{i} \text{for} \text{all} i \end{matrix}$$

where *i* indexes reactions and *U*_glucose_*=10*. The dual to this problem is:

$$\begin{matrix} \min_{r} \text{ }\sum_{i} U_{i}\cdot z_{i}\cdot r_{i} \\ S^{T}m-r=-c \\ r_{i}\leq\Omega_{i}\cdot\left(1-z_{i}\right) \text{for }i\neq\text{glucose} \\ r_{\text{glucose}}\geq0 \end{matrix}$$

where Ω_i_ represents a loose upper bound on the elements of the vector *r*, e.g. Ω_i_=1000. This dual formulation is linear when we treat *z* as a parameter “passed in” from the outer problem to the inner problem. However, the objective is nonlinear in the integrated single-level model, where we include *z* as a decision variable along with *r*.

Maranas’s lab has previously shown that they can resolve the nonlinearity in when *z* is included as a decision variable [[10](#_ENREF_10)]. However, the solution is highly non-trivial, and we are not aware of any publication that derives it. We infer that they resolved this nonlinearity by exploiting the fact that *U_i_z_i_r_i_=0* for all reactions *i* except *i=glucose*. *U_i_z_i_r_i_=0* for all these reactions, because *U_i_z_i_r_i_=0* when *z_i_=0* and when *z_i_=1*. If *z_i_*=0, then *U_i_z_i_r_i_*=0 trivially. If *z_i_=1* in , then the upper bound constraints on *v_i_* will not be tight, since *U_i_* is sufficiently large by design. Constraints that are not tight have dual variables of zero, so if *z_i_=1*, then *r_i_=0*, implying that *U_i_z_i_r_i_=0*. The reaction *i=glucose* is slightly different. This reaction is required, so we know it will be included in the model. Thus, we know that *z*_glucose_*=1*, so *z*_glucose_ no longer becomes a decision variable. Furthermore, because glucose is the limiting nutrient, we know that *v*_glucose_*=U*_glucose_*=10*, so its upper bound *U*_glucose_*=10* is tight. Tightness of this upper bound implies that *r*_glucose_*≥0*.

By exploiting the behavior of *U_i_z_i_r_i_*, program can be represented as,

$$\begin{matrix} \mathop{\text{min}}\limits_{m,r} \text{ }10\cdot r_{\text{glucose}} \\ S^{T}m-r=-c \\ r_{i}\leq\Omega_{i}\cdot\left(1-z_{i}\right) \text{for }i\neq\text{glucose} \\ r_{\text{glucose}}\geq0 \end{matrix}$$

Program does not possess the nonlinearity of program . Thus, program can be used to transform program into a single-level MILP, by transforming the inner problem into a set of constraints.

$$\begin{matrix} \min_{z} weights^{T}z \\ v_{\text{biomass}}=10r_{\text{glucose}} \\ Sv=0 \text{(inner} \text{problem)} \\ 0\leq v_{i}\leq U_{no-growth, i}\cdot z_{i} \\ S^{T}m-r=-c \\ r_{i}\leq\Omega_{i}\cdot\left(1-z_{i}\right) \text{for }i\neq\text{glucose} \\ r_{\text{glucose}}\geq0 \\ v_{\text{biomass}}\leq\text{minimal\_growth} \\ Sw=0 \\ 0\leq w_{i}\leq U_{wt, i}\cdot z_{i} \\ w_{\text{biomass}}\geq\text{minimal\_growth} \\ w_{\text{ATP}}\geq\text{atp\_maintenance} \\ z\in\{0,1\} \end{matrix}$$

## GrowMatch

The main difference in these conditions between the generic solution strategy given in program and GrowMatch [[6](#_ENREF_6),[12](#_ENREF_12)] concerns how *minimal_growth* is obtained and what its outer problem minimizes. The generic solution strategy constrains *v*_biomass_≤ *minimal_growth*, and its outer problem minimizes a weighted sum of reactions. Instead, GrowMatch’s outer problem minimizes growth, and it constrains the number of gene/reaction removals. This implies that GrowMatch again treats all reactions as having the same weight. In their original paper, they constrained the number of reaction removals per condition to be three [[6](#_ENREF_6)]. In their subsequent paper, they constrained the number of gene/reaction removals per condition to be two [[12](#_ENREF_12)].

## Model SEED

The main difference in these conditions between the generic solution strategy given in program and the Model SEED [[7](#_ENREF_7),[8](#_ENREF_8)] is the formulation of the dual in the inner problem. The Model SEED’s dual to FBA has an unexpected form.

Putting their dual to FBA into our nomenclature, we get:

$$\begin{matrix} \min_{m,r} \text{ }0^{T}m+U^{T}r \\ \left[\begin{matrix} S I \\ 0 -I \\ 0 I \end{matrix}\right]\left[\begin{matrix} m \\ r \end{matrix}\right]\geq\left[\begin{matrix} K\left(z-1\right)+c \\ -Kz \\ 0 \end{matrix}\right] \end{matrix}$$

where *K* is a large positive constant (e.g. 10,000). Since program is meant as the dual of FBA, we expect *its* dual to be FBA. For comparison, FBA for irreversible reactions is given in program . However, when we calculate the dual of program , we find:

$$\begin{matrix} \max_{v,\tau,\sigma} \text{ }v_{\text{biomass}}+Kv\left(z-1\right)-K\tau z \\ Sv=0 \\ v-\tau+\sigma=U \\ v,\tau,\sigma\geq0 \end{matrix}$$

where τ and σ are slack variables, *z* is treated as a parameter that is “passed in” from the outer problem, and *v*_biomass_*=c^T^v*. Program is not FBA.

This is a problem, because the Model SEED constrains the objective value of program to be equal to the objective value of FBA, whereas the strong duality theorem of linear programming equates the objective value of program and the objective value of program . Thus, if the objective value of program differs from the objective value of FBA, then the Model SEED’s MILP is infeasible.

We refer to program as a softly-constrained version of FBA. To see that it’s softly-constrained, consider the case where only a single reaction *i* is removed. This is represented as *z_i_=0*. In FBA, shown in program , we have *0≤v*_i_*≤U*_i_*z*_i_, so *v*_i_*=0*. However, in this case, program becomes:

$$\begin{matrix} \max_{v,\tau,\sigma} \text{ }v_{\text{biomass}}-Kv_{i}-K\sum_{j\neq i} \tau_{j} \\ Sv=0 \\ v-\tau+\sigma=U \\ v,\tau,\sigma\geq0 \end{matrix}$$

Now *v*_i_ can be positive if the objective pays a penalty of *Kv*_i_. So *z_i_=0* acts as a soft constraint. Now, consider a single reaction *j* with *z*_j_*=1*. Similarly, we see that *v*_j_ can exceed its upper bound *U*_j_ by τ_j_ if the objective pays a penalty of *K*τ_j_. So *U*_j_ also acts as a soft constraint. Thus, program treats the constraint *v≤Uz* as a soft constraint in general.

To characterize when *v*>*Uz*, consider the case where increasing the flux through a single reaction *l* from *v*_l_=*U*_l_*z*_l_ to *v*_l_=*U*_l_*z*_l_+Δ*v*_l_ increases growth by Δ*v*_biomass_. Then, we know that program will penalize the objective by *K*Δ*v*_l_. Thus, it will only be optimal for *v*_l_ to increase if Δ*v*_biomass_>*K*Δ*v*_l_, or equivalently if Δ*v*_biomass_/Δ*v*_l_>*K*. This inequality represents a small additional flux substantially adding to growth.

It is unclear how many reactions in the Model SEED’s database can have Δ*v*_biomass_/Δ*v*_l_>*K*. In our model, we see Δ*v*_biomass_/Δ*v*_l_*>100*, for example with MYO-INOSITOL-1-PHOSPHATE-SYNTHASE. This reaction has a large Δ*v*_biomass_/Δ*v*_l_, because myo-inositol is needed in small quantities for growth.

The possibility of Δ*v*_biomass_/Δ*v*_l_>*K* argues for increasing *K* in program . The problem with increasing *K*, though, is that optimization solvers have limited numerical precision, and large numbers in an optimization can have detrimental effect on the precision of small ones. However, difficulties with numerical precision of fluxes with high Δ*v*_biomass_/Δ*v*_l_ is not limited to the formulation in program . In fact, we had trouble ensuring that the constraint *v≤Uz* was enforced strictly, because solvers typically allow constraints to be violated by a small margin, such as 10^-6^.

We emphasize that this discussion does not nullify any of the Model SEED’s wonderful results; rather, it suggests that fully automated model reconstruction pipelines can still improve.

## CROP

There are three main differences in these conditions between the generic solution strategy given in program and CROP. The differences concern CROP’s (A) robustness to unbalanced reactions, (B) its smaller size, and (C) its ability to handle multiple conditions simultaneously.

**A. Robustness**

CROP was designed to be robust to unbalanced reactions, because we had unbalanced reactions in our database of metabolic reactions. These unbalanced reactions arose because of difficulties in exporting the MetaCyc reaction to text files, or because a reaction was unbalanced in MetaCyc. Other metabolic reaction databases, such as the Model SEED’s biochemistry database and KEGG, also have unbalanced reactions.

Although the existence of such unbalanced reactions is known [[13](#_ENREF_13)], we are not aware of any reconstruction algorithm that accounts for them when trying to suppress *in silico* growth. It was not feasible to manually balance all of the unbalanced reactions in our database. Instead, we had to select which of these reactions may belong in our final model.

We could not use previous algorithms to select among these unbalanced reactions. To see this, consider the *Generic solution strategy*. It assumes that the only upper bounds for FBA are those on limiting nutrients, such as glucose. However, an unbalanced reaction could have the form: water → glucose, and therefore act like the limiting nutrient. Because this unbalanced reaction wouldn’t have an upper bound according to the *Generic solution strategy*, its FBA would be unbounded. (Furthermore, we could not treat these reactions like nutrient sources, because doing so implies that we wouldn’t have any decision variable associated with these reactions, so wouldn’t be able to select which belonged in the final model.)

For CROP to be robust to unbalanced reactions, we developed a new version of FBA. This version of FBA is called Digital Unlimited-Nutrient Growth FBA (dung-FBA). dung-FBA is never unbounded, no matter how many unbalanced reactions there are, because it imposes an upper bound on growth: v_biomass_≤1. In fact, this is dung-FBA’s only upper bound. dung-FBA allows unlimited uptake of the available nutrients, so that the only factor limiting growth is connectivity. This feature is similar to OnePrune. Moreover, this allows dung-FBA to yield a binary (digital) result to the question: is growth possible? Mathematically, dung-FBA is defined by the following linear program:

$$\begin{matrix} \max_{v} c^{T}v=v_{\text{biomass}} \\ Sv=0 \text{(dung-FBA)} \\ 0\leq v \\ v_{biomass}\leq1 \end{matrix}$$

For CROP’s MILP, CROP requires the dual of dung-FBA. This dual is:

$$\begin{matrix} \min_{m,r} \text{ }r_{\text{biomass}} \\ S^{T}m-r=-c \text{(dual of dung-FBA)} \\ r_{\text{biomass}}\geq0 \\ r_{i}\leq0 \text{for }i\neq\text{biomass} \end{matrix}$$

where *r* is the dual variable associated with *r*eaction constraints, and *m* is the dual variable associated with *m*etabolite constraints. Now we add binary decision variables, *z*, so that we can select reactions to remove. dung-FBA now becomes:

$$\begin{matrix} \max_{v} c^{T}v=v_{\text{biomass}} \\ Sv=0 \\ 0\leq v_{i}\leq U_{i}\cdot z_{i} \text{for all }i \end{matrix}$$

where *U*_biomass_=1. The dual of this program is:

$$\begin{matrix} \min_{m,r} \text{ }r_{\text{biomass}} \\ S^{T}m-r=-c \\ r_{\text{biomass}}\geq0 \\ r_{i}\leq\Omega_{i}\cdot(1-z_{i}) \text{for }i\neq\text{biomass} \end{matrix}$$

For reactions *i* that are knocked out as a result of a gene knockout, *z*_i_ is treated as 0, implying *r*_i_ *≤* Ω_i_. Since Ω_i_ is supposed to be a loose upper bound, we represented this as *r*_i_ being free.

We used dung-FBA along with limed-FBA by replacing the *S* in dung-FBA with limed-FBA’s *S*_limed_. Using dung-FBA successfully brought us robustness to unbalanced reactions.

**B. Size**

FBA maximizes growth, so its dual represents an upper bound on growth. Thus, to mathematically express the experimental observation of no-growth (or of growth below a threshold), we need the dual. However, it also follows that once we have the dual, we no longer need FBA. In fact, incorporating both FBA and its dual only provides value if we want to model an intermediate level of growth. This observation appears to have been overlooked. However, only using the dual substantially reduces the MILP’s size.

This observation is actually suggested by program in *Generic solution strategy*. Program includes the constraints *v*_biomass_*=10r*_glucose_ and it includes *v*_biomass_*≤minimal_growth*. Together, these constraints imply that *10r*_glucose_*≤minimal_growth*, and that we can ignore *v*_biomass_. When we remove FBA’s growth maximization from the inner problem of program , we get:

$$\begin{matrix} \min_{z} weights^{T}z \\ S^{T}m-r=-c \\ r_{i}\leq\Omega_{i}\cdot\left(1-z_{i}\right) \text{for }i\neq\text{glucose} \\ r_{\text{glucose}}\geq0 \\ 10r_{\text{glucose}}\leq\text{minimal\_growth} \\ Sw=0 \\ 0\leq w_{i}\leq U_{wt, i}\cdot z_{i} \\ w_{\text{biomass}}\geq\text{minimal\_growth} \\ w_{\text{ATP}}\geq\text{atp\_maintenance} \\ z\in\{0,1\} \end{matrix}$$

One issue with the *Generic solution strategy* is that when there are no solutions that reduce growth to *minimal_growth*, solutions that partially reduce growth are not reported. To overcome this issue, *r*_glucose_ can be moved to the objective.

$$\begin{matrix} \min_{z} weights^{T}z+B\cdot r_{\text{glucose}} \\ S^{T}m-r=-c \\ r_{i}\leq\Omega_{i}\cdot\left(1-z_{i}\right) \text{for }i\neq\text{glucose} \\ r_{\text{glucose}}\geq0 \\ Sw=0 \\ 0\leq w_{i}\leq U_{wt, i}\cdot z_{i} \\ w_{\text{biomass}}\geq\text{minimal\_growth} \\ w_{\text{ATP}}\geq\text{atp\_maintenance} \\ z\in\{0,1\} \end{matrix}$$

where the constant *B* balances (i) the model’s consistency with reaction *weights* *versus* (ii) consistency with growth phenotypes.

Now, we replace the dual of FBA with the dual of dung-FBA, which involves substituting *r*_glucose_ with *r*_biomass_:

$$\begin{matrix} \min_{z} weights^{T}(1-z)\text{+}B\cdot r_{\text{biomass}} \\ S^{T}m-r=-c \\ r_{i}\leq\Omega_{i}\cdot(1-z_{i}),\text{ for }i\neq\text{biomass} \text{(dual of dung-FBA)} \\ r_{\text{biomass}}\geq0 \\ Sw\text{=0} \\ 0\leq w_{i}\leq U_{wt, i}\cdot z_{i} \text{(FBA)} \\ w_{\text{biomass}}\geq\text{minimal\_growth} \\ w_{\text{ATP}}\geq\text{atp\_maintenance} \\ z\in\{0,1\} \end{matrix}$$

A numerical difficulty that arises is that although *z*_i_ is supposed to be binary, its value may differ from 0 or 1 by a small amount, such as by 10^-6^. Thus, even when *z*_i_ is supposed to be 1, *r*_i_ may not be forced to be negative, but may instead reach 10^-6^*Ω_i_. To mitigate the effects of such numerical difficulties, we had some success with using rather small Ω_i_ for all reactions, e.g. Ω_i_=1. We found that using such tight upper bounds also improved run-time, which is consistent with the findings of others [[14](#_ENREF_14)].

This section shows that CROP deals with conditions where growth is predicted but no growth is experimentally observed with a MILP that is much smaller than previous algorithms, allowing for more efficient solution.

**C. Handling multiple conditions simultaneously**

Facilitated by its robustness and smaller size, CROP can handle multiple conditions where no growth is experimentally observed simultaneously. For two conditions where no growth is experimentally observed, the MILP is similar to above, albeit with an additional dual.

Handling multiple conditions simultaneously allows for identification of model modifications that synergistically solve these conditions.

References

1. Arakaki A, Huang Y, Skolnick J (2009) EFICAz2: enzyme function inference by a combined approach enhanced by machine learning. BMC Bioinformatics 10: 107.

2. Jankowski M, Henry C, Broadbelt L, Hatzimanikatis V (2008) Group Contribution Method for Thermodynamic Analysis of Complex Metabolic Networks. Biophysical Journal 95: 1487-1499.

3. Fleming RM, Thiele I, Nasheuer HP (2009) Quantitative assignment of reaction directionality in constraint-based models of metabolism: Application to Escherichia coli. Biophysical Chemistry 145: 47-56.

4. Bar-Even A, Noor E, Flamholz A, Buescher J, Milo R (2011) Hydrophobicity and charge shape cellular metabolite concentrations. PLoS computational biology 7: e1002166.

5. Henry C, Jankowski M, Broadbelt L, Hatzimanikatis V (2006) Genome-Scale Thermodynamic Analysis of Escherichia coli Metabolism. Biophysical Journal 90: 1453-1461.

6. Kumar V, Maranas C (2009) GrowMatch: An Automated Method for Reconciling In Silico/In Vivo Growth Predictions. PLoS Comput Biol 5: e1000308.

7. Henry C, Zinner J, Cohoon M, Stevens R (2009) iBsu1103: a new genome-scale metabolic model of Bacillus subtilis based on SEED annotations. Genome Biology 10: R69.

8. Henry C, DeJongh M, Best A, Frybarger P, Linsay B, et al. (2010) High-throughput generation, optimization and analysis of genome-scale metabolic models. Nat Biotech 28: 977-982.

9. Mitchell J (2001) Restarting after Branching in the SDP Approach to MAX-CUT and Similar Combinatorial Optimization Problems. Journal of Combinatorial Optimization 5: 151-166.

10. Burgard A, Maranas C (2003) Optimization-based framework for inferring and testing hypothesized metabolic objective functions. Biotechnology and bioengineering 82: 670-677.

11. Boyd S, Vandenberghe L (2004) Convex Optimization: Cambridge University Press.

12. Zomorrodi A, Maranas C (2010) Improving the iMM904 S. cerevisiae metabolic model using essentiality and synthetic lethality data. BMC Systems Biology 4: 178.

13. Gevorgyan A, Poolman M, Fell D (2008) Detection of stoichiometric inconsistencies in biomolecular models. Bioinformatics 24: 2245-2251.

14. Kim J, Reed J (2010) OptORF: Optimal metabolic and regulatory perturbations for metabolic engineering of microbial strains. BMC Systems Biology 4: 53.
