# Supplementary material for: Reconstruction and Validation of a Genome-Scale Metabolic Model for the Filamentous Fungus Neurospora crassa Using FARM
Source: PLoS Comput Biol. 2013 Jul 18;9(7):e1003126. doi: 10.1371/journal.pcbi.1003126 (PMC3730674; doi:10.1371/journal.pcbi.1003126)
Supplement: Text S4 — Simulating the effect of oxygen limitation on xylose fermentation. (DOCX) [file pcbi.1003126.s012.docx]

Simulating the effect of oxygen limitation on xylose fermentation

As a saprophyte capable of simultaneous saccharification and fermentation, Neurospora has potential industrial application as a biofuels organism [[1](#_ENREF_1)]. In particular, Neurospora’s ability to convert cellulose and hemicellulose to ethanol has been well studied [[2-8](#_ENREF_2)]. Interestingly, it has been shown for many fungi, including Neurospora, that when the primary constituent of hemicellulose, xylose, is used as a carbon source, differing cofactor preferences in the first two steps of the xylose degradation pathway cause a system wide redox imbalance under conditions of limited oxygen [[9-13](#_ENREF_9)]. We sought to gain greater physiological insight into the effect of oxygen limitation on xylose fermentation by applying our model to predict the critical oxygen level at which ethanol production and yield is maximized for Neurospora. The xylose fermentation pathways of the model are shown in Figure S5. In the first step, xylose is reduced to xylitol via a xylose reductase that has a strong cofactor preference for NADPH (XR-NADPH) and a weaker preference for NADH (XR-NADH) [[14](#_ENREF_14)]. In the second step, xylitol is either excreted extracellularly or oxidized to xylulose via a NAD+-dependent xylitol dehydrogenase (XDH) [[15](#_ENREF_15)]. Xylulose is then phosphorylated and enters the pentose phosphate pathway, followed by glycolysis to pyruvate, and is either excreted as ethanol or enters the mitochondria where it is oxidized via the TCA [[10](#_ENREF_10)]. We simulated the effect of oxygen limitation on xylose fermentation by maximizing ATP production for different oxygen levels and imposing the measured XR-NADPH:XR-NADH ratio as a constraint between these two fluxes [[10](#_ENREF_10)]. Under anaerobic conditions, as shown in Figure S5a, our model predicts that XR-NADH is the primary source of NAD+ for XDH, so the excess xylitol generated by XR-NADPH is excreted, resulting in lower observed ethanol yields [[10](#_ENREF_10)]. Under fully aerobic conditions, as shown in Figure S5b, XDH no longer acts as a bottleneck because NAD+ is regenerated by oxygen via the glycerol phosphate shuttle, which explains why no xylitol is produced at high oxygen levels [[10](#_ENREF_10)]. Furthermore, enough oxygen is present to generate the NAD+ required by the TCA cycle, which converts all the pyruvate to CO2 and water, which explains why no ethanol production is observed at high oxygen levels [[10](#_ENREF_10)]. In Figure S5c, the model predicts the critical oxygen level where just enough NAD+ is regenerated via the glycerol phosphate shuttle to allow XDH to catalyze all the xylitol, but there is no NAD+ left over to power the TCA cycle, resulting in optimal ethanol yield and productivity**.** All three predictions of xylitol and ethanol yields are consistent with experimental observation [[10](#_ENREF_10)]

References

1. Znameroski EA, Glass NL (2013) Using a model filamentous fungus to unravel mechanisms of lignocellulose deconstruction. Biotechnol Biofuels 6: 6.

2. Sun J, Tian C, Diamond S, Glass NL (2012) Deciphering transcriptional regulatory mechanisms associated with hemicellulose degradation in Neurospora crassa. Eukaryot Cell 11: 482-493.

3. Tian C, Beeson WT, Iavarone AT, Sun J, Marletta MA, et al. (2009) Systems analysis of plant cell wall degradation by the model filamentous fungus Neurospora crassa. Proc Natl Acad Sci U S A 106: 22157-22162.

4. Xiros C, Topakas E, Katapodis P, Christakopoulos P (2008) Hydrolysis and fermentation of brewer's spent grain by Neurospora crassa. Bioresour Technol 99: 5427-5435.

5. Yazdi MT, Radford A, Keen JN, Woodward JR (1990) Cellulase production by Neurospora crassa: purification and characterization of cellulolytic enzymes. Enzyme Microb Technol 12: 120-123.

6. Deshpande V, Keskar S, Mishra C, Rao M (1986) Direct conversion of cellulose/hemicellulose to ethanol by Neurospora crassa. Enzyme and Microbial Technology 8: 149-152.

7. Rao M, Deshpande V, Keskar S, Srinivasan MC (1983) Cellulase and ethanol production from cellulose by Neurospora crassa. Enzyme and Microbial Technology 5: 133-136.

8. Eberhart B, Cross DF, Chase LR (1964) Beta-Glucosidase System of Neurospora Crassa. I. Beta-Glucosidase and Cellulase Activities of Mutant and Wild-Type Strains. J Bacteriol 87: 761-770.

9. Fromanger R, Guillouet SE, Uribelarrea JL, Molina-Jouve C, Cameleyre X (2010) Effect of controlled oxygen limitation on Candida shehatae physiology for ethanol production from xylose and glucose. J Ind Microbiol Biotechnol 37: 437-445.

10. Zhang Z, Qu Y, Zhang X, Lin J (2008) Effects of oxygen limitation on xylose fermentation, intracellular metabolites, and key enzymes of Neurospora crassa AS3.1602. Applied Biochemistry and Biotechnology 145: 39-51.

11. Meijer S, Panagiotou G, Olsson L, Nielsen J (2007) Physiological characterization of xylose metabolism in Aspergillus niger under oxygen-limited conditions. Biotechnol Bioeng 98: 462-475.

12. Vandeska E, Kuzmanova S, Jeffries TW (1995) Xylitol formation and key enzyme activities in Candida boidinii under different oxygen transfer rates. Journal of Fermentation and Bioengineering 80: 513-516.

13. Skoog K, Hahn-H√§gerdal Br (1990) Effect of Oxygenation on Xylose Fermentation by Pichia stipitis. Applied and Environmental Microbiology 56: 3389-3394.

14. Woodyer R, Simurdiak M, van der Donk WA, Zhao H (2005) Heterologous expression, purification, and characterization of a highly active xylose reductase from Neurospora crassa. Appl Environ Microbiol 71: 1642-1647.

15. Phadtare SU, Rawat UB, Rao MB (1997) Purification and characterisation of xylitol dehydrogenase from <i>Neurospora crassa</i> NCL communication No. 6347. FEMS Microbiology Letters 146: 79-83.
